# Novel Type I/II Carbazole/Benzindole Photosensitizers Achieve Chemo-Photodynamic Synergistic Therapy for Suppressing Solid Tumors and Drug-Resistant Bacterial Infections

**DOI:** 10.3390/molecules30122560

**Published:** 2025-06-12

**Authors:** Zihao Wang, Xiao Liu, Yifan Ma, Jiaxin Zheng, Ke Xu, Yingxue Chang, Zhaoyan Ye, Yong Ling, Lei Wang

**Affiliations:** 1 Department of Medicinal Chemistry, School of Pharmacy, Nantong University, Nantong 226001, China; bill1447838083@163.com (Z.W.); lx199803@126.com (X.L.); 2323320006@stmail.ntu.edu.cn (Y.M.); 19912127992@163.com (J.Z.); 15288077201@163.com (K.X.); 19603923217@163.com (Y.C.); jackcooper1128@163.com (Z.Y.); 2 Department of Traditional Chinese Pharmacy, China Pharmaceutical University, Nanjing 210009, China

**Keywords:** photodynamic therapy (PDT), drug-resistant bacterial infection, MRSA, reactive oxygen species (ROS), tumor microenvironment

## Abstract

To address the clinical challenges posed by symbiotic drug-resistant bacterial infections and tumor microenvironments, this study designed and synthesized novel carbazole/benzindole-based photosensitizers **A1**–**A4**, systematically evaluating their antitumor and antibacterial therapeutic potential through chemo-photodynamic therapy. Especially, compound **A4** demonstrated potent Type I/II reactive oxygen species (ROS) generation capabilities. In vitro experiments revealed that **A4** concentration-dependently inhibited HT-29 cells under hypoxic conditions (IC_50_ = 0.89 μM) with a prominent photodynamic index (PI > 9.23), and substantially promoted cancer cell programmed death. In antibacterial evaluations, **A4** achieved the complete eradication of dermal MRSA infections within 7 days through ROS-mediated membrane disruption under illumination. In the HT-29 xenograft model, the PDT–chemotherapy synergy strategy achieved a tumor suppression rate of 96%. This work establishes an innovative strategy for the combinatorial management of multidrug-resistant infections and solid tumors.

## 1. Introduction

Malignant tumors pose a severe threat to global health. Recent studies have revealed that Methicillin-resistant *Staphylococcus aureus* (MRSA) exhibits competitive niche advantages within tumor microenvironments [1,2]. Clinical evidence demonstrates a 19.8% MRSA infection rate among post-operative colorectal cancer patients, with MRSA colonization reducing HT29 cell sensitivity to oxaliplatin by 3.2-fold [3]. Mechanistic investigations indicate that MRSA significantly diminishes chemotherapeutic bioavailability through the membrane adsorption of 5-fluorouracil (5-FU), evidenced by a 41% reduction in AUC_0–24_ [4,5]. This tumor–microbe symbiosis fosters a vicious cycle of “infection-drug resistance-metastasis” [6,7]. Consequently, developing therapeutic agents capable of simultaneously targeting malignancies and resistant infections represents a critical strategy for improving cancer treatment outcomes.

The medical community has reached a dual consensus: On the one hand, monotherapeutic chemotherapy regimens exhibit inherent limitations in addressing bacterial biofilm-associated infections, as they fail to effectively penetrate and disrupt biofilm matrices, which is a critical deficit that necessitates multimodal combination strategies [8,9,10,11]. On the other hand, whilst conventional tri-modality therapy (surgery, radiotherapy, and chemotherapy) has established standardized clinical pathways in oncology, the intrinsic microenvironmental heterogeneity and regional hypoxia characteristic of solid tumors result in chemoresistance or radioresistance in 30–40% of cases [12,13].

Within this context, photodynamic therapy (PDT) demonstrates unique therapeutic value; its mechanism, mediated through photosensitizer-generated reactive oxygen species (ROS) under specific wavelength excitation [14,15,16], confers dual therapeutic merits: in antimicrobial applications, ROS effectively eradicate drug-resistant pathogens while degrading biofilm extracellular polymeric substances [17,18,19]; in oncology, PDT selectively disrupts tumor vasculature and induces cancer cell apoptosis [20,21,22]. Recent advances reveal that the spatiotemporal coordination of chemotherapeutics with PDT generates synergistic “1 + 1 > 2” therapeutic outcomes [23,24,25,26]. This chemo-photodynamic combinatorial approach not only enhances ROS cytotoxicity via pharmacological sensitization, but also enables precise spatial–temporal drug concentration control through photoactivated drug delivery systems, offering an innovative paradigm for combating resistant infections and solid tumors [27].

**Figure 1:**
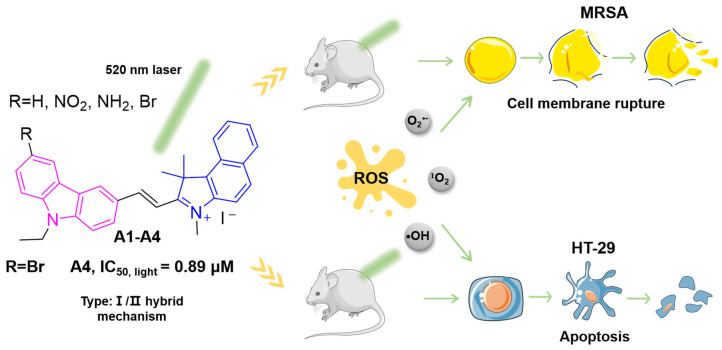
Chemical structures of novel carbazole/benzindole photosensitizers **A1**–**A4** and schematic diagram illustrating their ROS-mediated synergistic mechanisms: membrane disruption in MRSA and pro-apoptotic activity in tumor cells.

Despite PDT’s demonstrated advantages in antibacterial and antitumor applications, the current lack of ideal dual-functional photosensitizers combining potent antibacterial efficacy with tumor-targeting specificity necessitates the development of advanced photosensitizing agents [28,29]. Notably, quaternization design strategies enhance antimicrobial activity, as quaternary ammonium salts are widely employed for their biofilm-eradicating activity and membrane-targeting mechanisms [30,31,32]. Herein, benzindolium quaternary salts were utilized as strong electron acceptors, coupled with carbazole tricycles as auxiliary electron-withdrawing moieties, to design and synthesize novel carbazole/benzindole-based photosensitizers **A1**–**A4** (Figure 1) [33,34,35]. Carbazole derivatives further exhibit multi-target engagement capabilities, including interactions with DNA and DNA gyrase. We anticipate that these engineered photosensitizers will achieve synergistic therapeutic effects against malignancies and MRSA through robust chemo-photodynamic integration.

## 2. Results and Discussion

### 2.1. Synthesis and Characterization

**Scheme 1:**
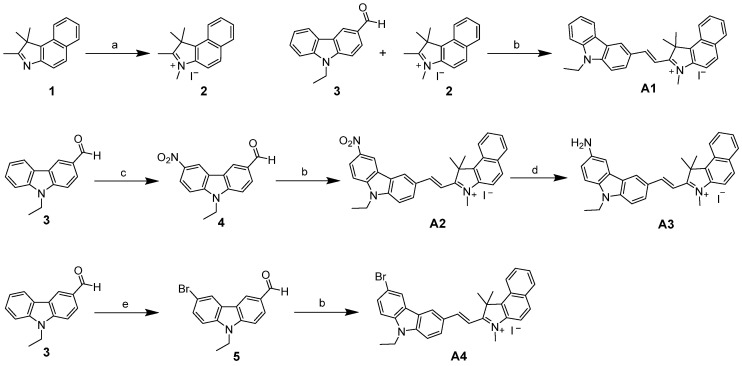
Synthetic route. (a) CH_3_I, CH_3_CN, 80 °C (b) C_5_H_11_N, ethanol, 80 °C, and 60–75% (c) CH_3_COOH, HNO_3_, 0 °C, and 85% (d) Fe, NH_4_Cl, 80 °C, and 55%, and (e) NBS, TBAB, 65 °C, and 85%.

The synthetic pathway for target compounds is delineated in Scheme 1. The initial quaternization of benzindole **1** with iodomethane yielded benzindolium salt **2**, which subsequently underwent Knoevenagel condensation with carbazole-3-carbaldehyde **3** to afford compound **A1**. For **A2** synthesis, nitro group installation at the C6 position of **3** generated intermediate **4**, followed by Knoevenagel condensation and subsequent iron-mediated reduction to produce **A3**. The bromination of **3** using NBS furnished intermediate **5**, and finally, it was condensed with phenylindole iodized salt to obtain the target compound **A4**. All target compounds exhibited > 95% purity (Figures S24–S27), with structural verification achieved through ^1^H NMR, ^13^C NMR, and ESI-HRMS analyses, as detailed in Supplementary Data (Figures S12–S23).

### 2.2. Determination of Photodynamic Properties

**Figure 2:**
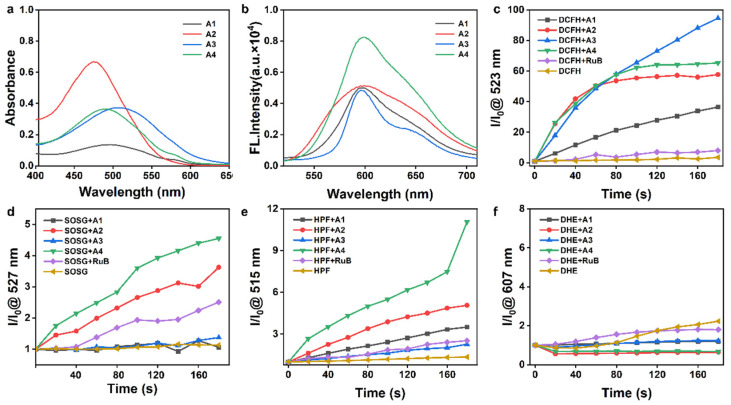
UV–Vis (**a**) and fluorescence spectra (**b**) of compounds **A1**–**A4** (10 µM) in deionized water at room temperature. (**c**) DCFH for detecting ROS, (**d**) SOSG reagent for ^1^O_2_, (**e**) HPF for •OH, and (**f**) DHE for O_2_^•−^ detection across various groups. The time interval for irradiation time t: 20 s. Light source: 520 nm, 50 mW/cm^2^.

First, the photophysical properties of compounds **A1**–**A4** were characterized using UV-Vis spectrophotometry and fluorescence spectroscopy. Four target compounds exhibited characteristic absorption bands within the 475–510 nm visible light range, with corresponding fluorescence emission maxima consistently centered at approximately 600 nm (Figure 2a,b).

PDT, a clinically validated antitumor modality, operates through photosensitizer (PS)-generated ROS under light activation to achieve targeted tumor cell eradication [36]. We used DCFH probes to detect the ROS generation levels of different photosensitizers under light treatment [36]. The experimental data revealed superior ROS induction capabilities in the **A1**–**A4** compared to the in conventional photosensitizer [Ru(bpy)_3_]^2+^ (RuB), with **A3** exhibiting the highest ROS generation, followed by **A4** and **A2** (Figure 2c and Figure S1).

Next, complementary singlet oxygen (^1^O_2_) detection via SOSG assay demonstrated significantly enhanced ^1^O_2_ production in **A4** and **A2** relative to RuB (Figure 2d and Figure S2). Notably, no discernible superoxide anion (O_2_^•−^) generation was detected across any compounds using dihydroethidium probes (Figure 2f and Figure S4). Intriguingly, hydroxyl radical (•OH) accumulation was specifically observed in **A4**-treated samples under illumination via HPF probe detection (Figure 2e and Figure S3).

The above data show that **A4** exhibits outstanding photosensitizing properties, likely exerting therapeutic effects in hypoxic tumor microenvironments through Type I/II photodynamic mechanism, and it is worthy of in-depth development as a new candidate molecule for PSs.

### 2.3. Electronic Spin Resonance Detection

We further investigated the photodynamic properties of active photosensitizer **A4** using electron spin resonance (ESR) spectroscopy. Under illumination, distinct ESR signals of •OH, O_2_^•−^, and ^1^O_2_ were detected, whereas no significant signals were observed in the dark (Figure S6). These results align with photodynamic assays, confirming **A4**’s superior Type I/II PDT capability. During photoexcitation, electrons transition from the ground state (S_0_) to higher-energy excited states (S_n_), followed by internal conversion to the lowest singlet excited state (S_1_). The S_1_ state either returns radiatively to S_0_ (fluorescence emission) or undergoes intersystem crossing (ISC) to the triplet state (T_1_). The T_1_ state generates O_2_^•−^/•OH via electron transfer (Type I) or ^1^O_2_ via energy transfer (Type II). Efficient ISC is a critical determinant of ROS generation by photosensitizers (PSs). The results show that **A4** has a narrow HOMO-LUMO energy gap, high **ΔE_(LUMO-HOMO)_** value (**ΔE_(LUMO-HOMO)_** = 2.73 eV), and low ΔE_ST_ (1.14 eV) (Figure S7). These electronic characteristics underscore **A4**’s exceptional ROS-generating efficiency under photoactivation.

### 2.4. Chemotherapy/Photodynamic Antitumor Activity

**Figure 3:**
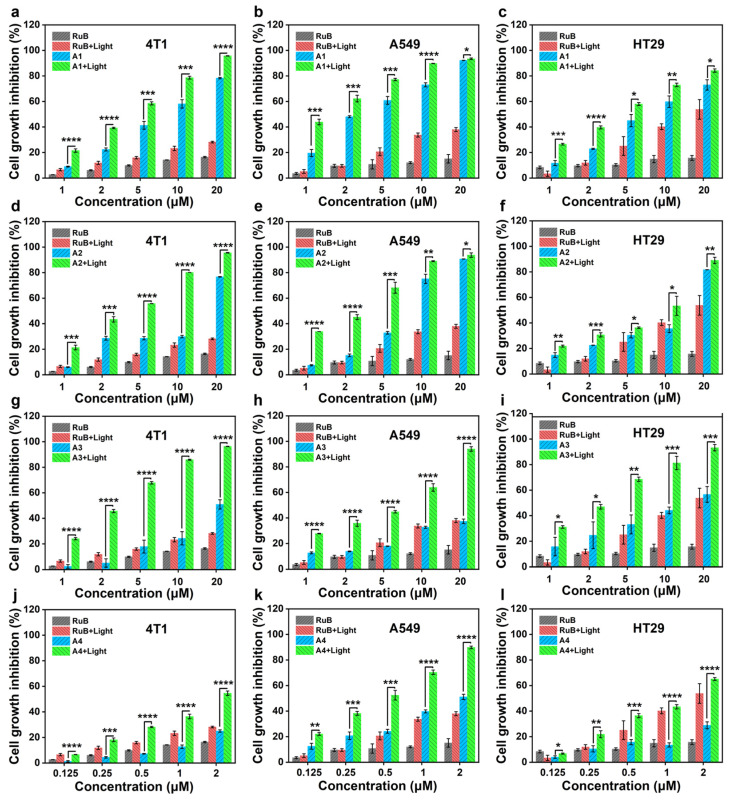
Inhibitory activities on 4T1 (**a**,**d**,**g**,**j**), A549 (**b**,**e**,**h**,**k**), and HT29 cells (**c**,**f**,**i**,**l**) exhibited by RuB and **A1**–**A4** with/without irradiation, L: light. Light source: 520 nm, 100 mW/cm^2^, 10 min, mean ± SD, n = 3, * *p* < 0.05, ** *p* < 0.01, *** *p* < 0.001, and **** *p* < 0.0001.

Building on the reported pharmacodynamic advantages of natural carbazole scaffolds in chemotherapy [37] and the exceptional ROS-inducing properties of compounds **A4** and **A2** identified in this study, we systematically evaluated the antitumor efficacy of this series against multiple cancer cell lines (human lung cancer A549, human colon cancer HT-29, and murine breast cancer 4T1) under chemo-photodynamic combination therapy, using [Ru(bpy)_3_]^2+^ (RuB) as the positive control. Under normoxic conditions, **A1**, **A2**, and **A3** demonstrated significant antitumor activity compared to RuB, but exhibited limited photodynamic effects upon illumination (Figure 3a–i). Notably, **A4** showed low activity in the dark (IC_50_ < 100 μM for HT-29, Table S1), yet achieved remarkable light-enhanced antitumor activity (photodynamic index PI > 9.23), highlighting its superior photodynamic therapeutic potential (Figure 3j–l). Given **A4**’s outstanding synergistic antitumor performance and Type I ROS generation capacity, we further investigated its efficacy against HT-29 cells under hypoxic conditions. **A4** effectively inhibited HT-29 cells in a concentration-dependent manner (IC_50,light_ = 0.87–0.91 μM, Table S1; Figure S5), confirming its hypoxia-compatible therapeutic potential through combined chemo-photodynamic action. Notably, **A4** outperformed other compounds in the series, particularly in photodynamic efficacy under hypoxic tumor microenvironments.

### 2.5. Determination of ROS Generation

**Figure 4:**
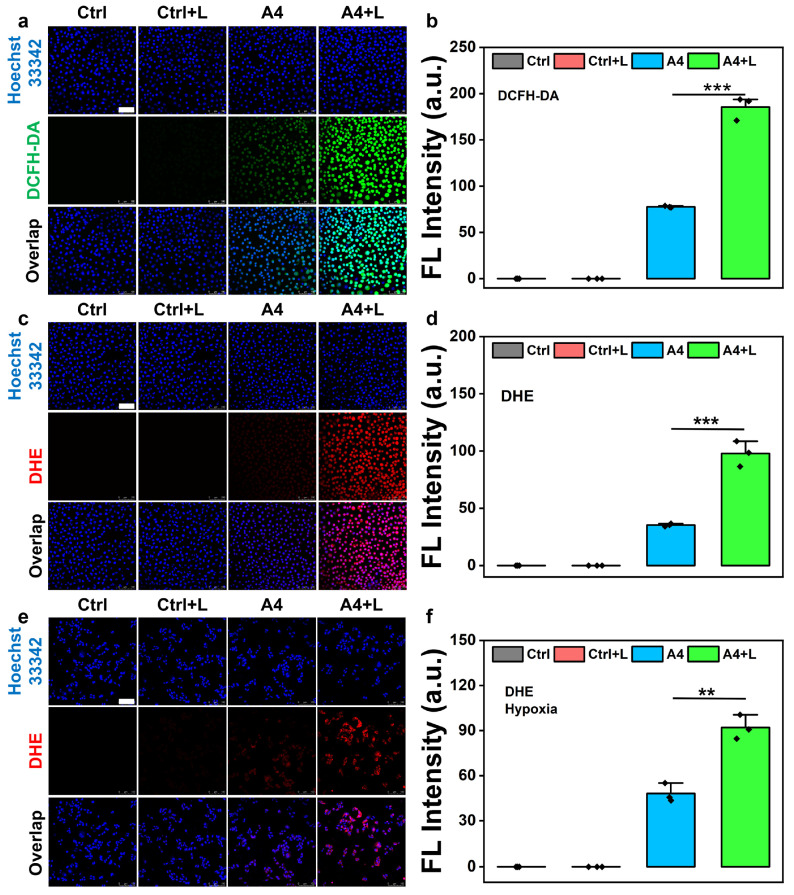
(**a**) Fluorescence microscopic imaging of HT29 cells in different groups after treatment with DCFH-DA. (**b**) Average fluorescence intensity of DCFH-DA of HT29 cells in different groups. (**c**–**f**) Fluorescence microimaging of 4T1 cells in different groups treated with DHE in (**c**) normoxic or (**e**) hypoxic environments. Average fluorescence intensity of DHE in HT29 cells of different groups in (**d**) normoxic or (**f**) hypoxic environments. L: light. Light source: 520 nm, 100 mW/cm^2^, 10 min, scale bar = 100 μm, mean ± SD, n = 3, ** *p* < 0.01, and *** *p* < 0.001.

The results of the MTT assay showed that compound **A4** exhibited good antitumor cell activity. Based on this, we investigated whether **A4** could induce ROS production in cancer cells using 2′,7′-dichlorodihydrofluorescein diacetate (DCFH-DA) to measure the intracellular ROS levels. Significant fluorescence signals were observed in **A4**-treated cells, while PBS and PBS + light (L) treatments did not induce any notable fluorescence (Figure 4a,b). Additionally, strong green fluorescence was detected in **A4**-pretreated cells after light irradiation, indicating that illumination further enhanced the ROS levels. To visualize Type I PDT effects triggered by **A4**, intracellular O_2_^•−^ levels were evaluated using the superoxide indicator dihydroethidium (DHE). Cells co-treated with **A4** and DHE exhibited intense red fluorescence after illumination, whereas non-irradiated cells showed weaker fluorescence, demonstrating increased O_2_^•−^ generation post illumination (Figure 4c,d). Considering the low oxygen dependency of O_2_^•−^ production in Type I PDT, we also assessed intracellular O_2_^•−^ generation under hypoxic conditions. **A4** and **A4** + L co-treated cells still displayed bright DHE signals in hypoxia (Figure 4e,f), confirming that **A4** can execute hybrid Type I/II PDT by generating ROS even under low oxygen conditions.

### 2.6. Live/Dead Cell Staining

Additionally, live/dead staining assays were conducted on HT29 cells to evaluate the efficacy of **A4** in promoting cancer cell death, particularly its enhanced effects under laser irradiation. The results revealed that **A4**-treated cells without laser irradiation exhibited a certain mortality rate, indicating that **A4** itself possesses certain chemotherapeutic effects capable of directly inducing partial cancer cell death. However, when **A4**-treated cells were subjected to laser irradiation, the number of dead cells significantly increased, demonstrating that **A4** under laser irradiation markedly enhances its ability to induce cell death (Figure S8). This enhancement is attributed to the synergistic effects of **A4**’s chemotherapeutic activity and PDT, where **A4** generates ROS under laser irradiation, further exacerbating cell death. Furthermore, MTT assay results were consistent with the live/dead staining data, confirming that **A4** significantly reduces cell viability under laser irradiation. These findings suggest that **A4** exerts significant antitumor effects through the synergy of chemotherapy and PDT under laser irradiation, providing robust experimental support for **A4** as a novel photodynamic therapeutic agent.

### 2.7. Study of In Vitro Antimicrobial Capacity

**Figure 5:**
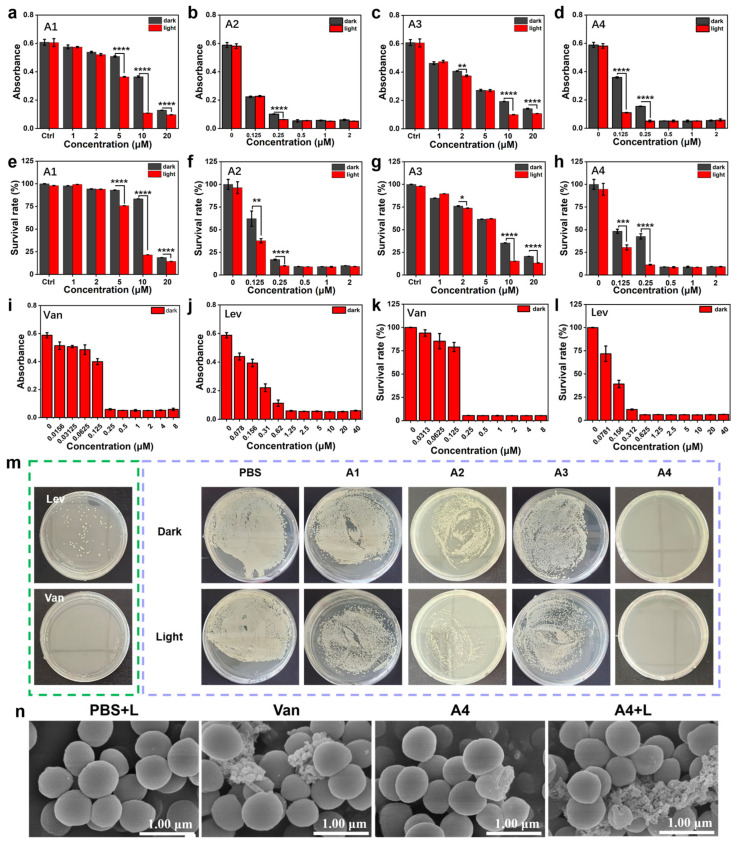
Anti-MRSA (**a**–**d**,**i**,**j**) (original OD_600_ = 0.6) performance of **A1**–**A4** and (**e**–**h**,**k**,**l**) the bacteriostatic efficacy of **A1**–**A4** was determined through the CCK-8 gradient dilution technique, with vancomycin and levofloxacin serving as the control agents. (**m**) The efficacy of plate streaking for treating MRSA with/without irradiation. Concentration: 1 μM. (**n**) The SEM images of MRSA subjected to various treatment groups. L: light. Light source: 520 nm, 100 mW/cm^2^, 5 min, scale bar = 200 nm, mean ± SD, n = 3, * *p* < 0.05, ** *p* < 0.01, *** *p* < 0.001, and **** *p* < 0.0001.

The antibacterial activity of **A1**–**A4** against MRSA was first evaluated using a turbidimetric assay, with vancomycin (Van) and levofloxacin (Lev) as positive controls. As shown in Figure 5a–l, after treating MRSA with compounds of different concentrations under light conditions, observe the OD value at 600 nm; **A1** and **A3** exhibited weak anti-MRSA effects at various concentrations. Compared with **A1**, **A3**, and **A4**, **A2** demonstrated negligible photodynamic antibacterial activity, and **A4**-treated MRSA displayed obvious concentration-dependent antibacterial activity characteristics (Figure 5d). The CCK-8 bacterial viability assay was further employed to validate the in vitro antibacterial activity of **A1**–**A4** under light irradiation, with dark conditions as the control. The results were consistent with the turbidimetric assay, showing concentration-dependent antibacterial effects under light irradiation. At 1 μM under light irradiation, **A4** exhibited potent antibacterial efficacy, comparable to Van at 1 μM, and demonstrated activity similar to Lev at 1.25 μM, while dark conditions showed no significant impact on bacterial growth (Figure 5e–h,k,l). These results indicate that ROS generated using **A4** under light irradiation contribute to its potent antibacterial activity. Subsequently, a plate spread assay was used to quantitatively assess the bactericidal capacity of **A1**–**A4** against MRSA. Colony counting analysis revealed that **A4**, when co-incubated with MRSA under light irradiation, significantly inhibited colony growth compared to other controls, demonstrating enhanced synergistic bactericidal effects between PDT and **A4** (Figure 5m). Next, the DCFH-DA fluorescent probe was utilized to evaluate ROS production in bacteria treated with **A4** combined with laser irradiation. The results showed intense green fluorescence in the **A4** + L group, whereas control groups exhibited no detectable fluorescence signals (Figure S9). This confirms the robust ROS-generating capability of **A4** under light irradiation at the bacterial level. Finally, to further validate the damaging effect of **A4** on bacterial membranes, scanning electron microscopy (SEM) was employed to observe the morphological changes in MRSA treated with **A4**, as shown in Figure 5. After subjecting cultured MRSA to different treatments, untreated MRSA under laser irradiation exhibited plump bacterial morphology with smooth and intact surfaces. In contrast, Van-treated and **A4**-treated (dark) MRSA displayed rough, ruptured surfaces and overall structural damage, indicating partial bactericidal effects. Notably, **A4** combined with laser irradiation caused pronounced wrinkling of bacterial membrane surfaces and significant peripheral cellular debris, signifying membrane rupture and bacterial lysis. These results confirm that **A4** and PDT synergistically exert antibacterial effects (Figure 5n).

### 2.8. In Vivo Antimicrobial Effect

**Figure 6:**
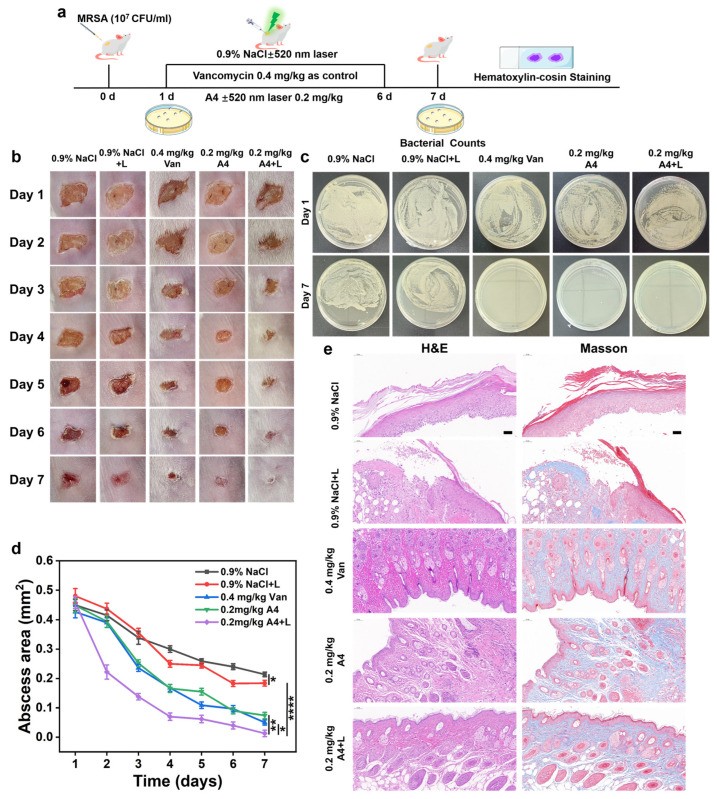
(**a**) Establishment of a murine skin defect wound model infected with MRSA (**b**) the assessment of wound healing, (**c**) plate counting images of bacteria extracted from wounds, (**d**) quantitative analysis of wound area, and (**e**) histopathological evaluation via H&E and Masson staining across various treatment groups. L: light. Light source: 520 nm, 100 mW/cm^2^, 5 min, scale bar = 50 μm, mean ± SD, n = 3, * *p* < 0.05, ** *p* < 0.01, and **** *p* < 0.0001.

Next, the in vivo antibacterial efficacy of **A4** will be further studied. We established a MRSA-infected skin defect wound model in mice (Figure 6a). ICR mice were selected as experimental subjects. After anesthesia with isoflurane, the dorsal skin was shaven, cleaned, and disinfected with 75% ethanol. A 1 × 1 cm^2^ surgical wound was created on the back, followed by infection with MRSA suspension (10^7^ CFU/mL). The wound infection model was successfully established 24 h later. Infected mice were randomly divided into five groups (n = 5 per group): 0.9% NaCl, 0.9% NaCl + L, **A4** (0.2 mg/kg), **A4** + L, and vancomycin (0.4 mg/kg) as the positive control. Drugs were topically applied to the wounds, with the laser-irradiated groups receiving 200 mW/cm^2^ irradiation for 10 min. The wound healing progression was photographically documented daily for 7 days post treatment, and the wound areas were quantified to evaluate the therapeutic outcomes. As shown in Figure 6b, control group mice developed severe abscesses (~50 mm^2^) on dorsal skin, whereas vancomycin (0.4 mg/kg) and **A4** (0.2 mg/kg) treatments led to scab formation at infection sites. Notably, **A4** + L (0.2 mg/kg) treatment significantly reduced abscess size. To further assess antibacterial efficacy, bacteria were harvested from wounds on days 1 and 7 post treatment for plate counting. MRSA colony counts markedly decreased in vancomycin- (0.4 mg/kg) and **A4**-treated (0.2 mg/kg) groups, while no colonies were observed in the **A4** + L group, indicating complete bacterial eradication (Figure 6c).

Additionally, to more intuitively evaluate the effect of **A4** on infected wounds under light conditions, a semiquantitative analysis of wound areas across treatment groups was performed. The results showed that on day 7 post treatment, the wound area in the **A4** + L group decreased to 0.03% of the original size, whereas the 0.9% NaCl and vancomycin groups exhibited final wound areas of 0.06% and 0.26%, respectively. This indicates that **A4** combined with laser irradiation significantly enhanced anti-MRSA activity, demonstrating greater potential than vancomycin (Figure 6d). Further histological examinations with H&E and Masson staining were conducted on skin tissues. As shown in Figure 6e, after 7 days of treatment, H&E staining revealed extensive cellular voids, disrupted tissue integrity, increased inflammatory cell infiltration, and thickened epidermal layers in the 0.9% NaCl and 0.9% NaCl + L groups after MRSA infection. Vancomycin treatment reduced inflammatory cell infiltration. In the **A4** group, wound sections displayed mostly healed tissue with vigorous cellular growth, though partial gaps and residual cell loss persisted. In contrast, the **A4** + L group exhibited complete tissue restoration with normal architecture, no cellular defects, newly formed hair follicles, and markedly reduced inflammatory infiltration. These findings confirm the superior wound healing efficacy of **A4** + L compared to the control groups.

To further analyze the wound healing activity of **A4**, we employed Masson staining to assess collagen degradation and deposition at inflammatory sites. Results revealed that the **A4** + L group exhibited densely structured collagen fibrils (stained blue), with the highest collagen deposition in the dermis and thickened collagen fibers by day 7. In contrast, the control groups showed predominantly red-stained collagen fibrils, suggesting residual collagen degradation in these groups. These findings demonstrate that ROS generated using **A4** under light conditions effectively treat MRSA skin infections and promote tissue remodeling and regeneration in mice.

### 2.9. In Vivo Antitumor Assessment

**Figure 7:**
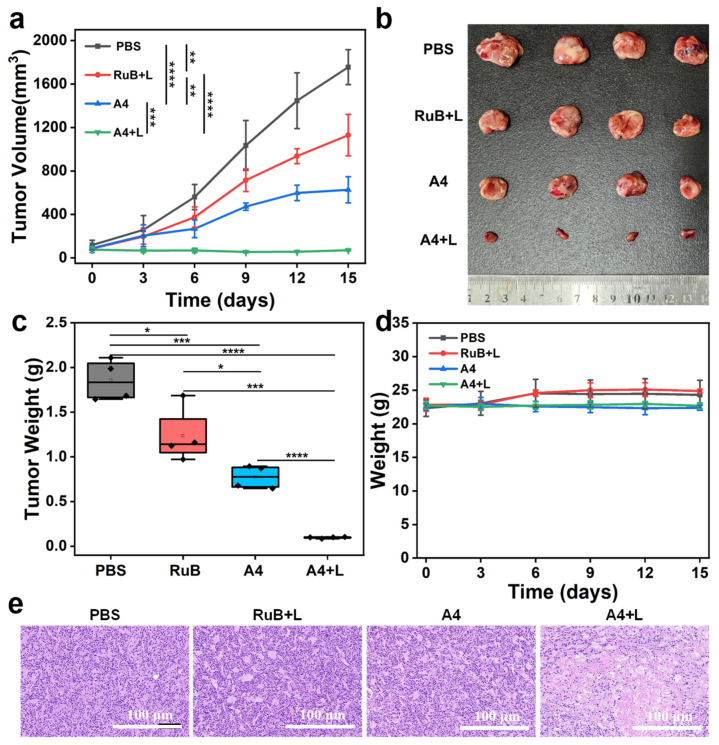
(**a**) Tumor growth curves of the tumor-bearing mice with various treatments, (**b**) images of tumors harvested from tumor-bearing mice at 15 days after different treatments. (**c**) Tumor weights of mice after different treatments, (**d**) body weight changes in HT29 tumor-bearing mice with different treatments, and (**e**) H&E staining of tumor tissue sections collected from mice in different groups at the end of the treatment (scale bar = 100 μm). L: light. Light source: 520 nm, 100 mW/cm^2^, 10 min, and mean ± SD, n = 3. * *p* < 0.05, ** *p* < 0.01, *** *p* < 0.001, and **** *p* < 0.0001.

Given **A4**’s significant ability to promote ROS generation in tumor cells and its potent photodynamic antitumor efficacy, we further evaluated its in vivo chemo-photodynamic therapy performance in an HT-29 tumor-bearing mouse model. When tumor volumes reached approximately 80 mm^3^, mice were randomized into four groups (n = 4 per group): PBS control, RuB + L, **A4** alone, and **A4** + L, and the drug was administered via intratumoral injection at a dose of 10 mg/kg every three days for a total of five times. Tumor volumes and body weights were measured every 3 days over a 15-day treatment period. The results demonstrated rapid tumor growth in the PBS and RuB + L groups. In contrast, the **A4** group exhibited significantly slowed tumor progression, achieving an inhibition rate of 64.3%, far surpassing the RuB control (35.7% inhibition) and likely attributed to **A4**’s inherent chemotherapeutic activity. Notably, **A4** combined with laser irradiation (**A4** + L) induced remarkable tumor suppression, with an inhibition rate of 96.0%, significantly outperforming **A4** alone and underscoring the efficacy of chemo-photodynamic synergy (Figure 7a). Upon completion of the study, the excised tumor tissues were weighed. Compared to the PBS group, **A4** + L reduced tumor weight by 94.7%, whereas RuB + L and **A4** alone achieved reductions of 33.4% and 58.3%, respectively (Figure 7b,c).

Furthermore, no significant changes in body weight were observed across any group during the treatment period (Figure 7d). At the end of the study, major organs and tumor samples from each group were collected for hematoxylin and eosin (H&E) staining. Histological evaluation revealed no apparent pathological damage in organ samples, with tissue sections maintaining the normal cellular morphology and architecture (Figure S10). H&E-stained tumor sections from the **A4** + L group exhibited widespread nuclear pyknosis and enhanced cytoplasmic eosinophilia, indicative of therapy-induced antitumor effects. Serum biochemical analysis further demonstrated that the liver function markers (ALT/AST) and renal function indicators (CREA/UREA) remained within the normal ranges, showing no statistically significant differences compared to the PBS control group (Figure S11). Combined with histopathological and blood biochemical results, these data suggest the negligible systemic toxicity of **A4**. These findings underscore that **A4** not only exhibits robust in vivo antitumor efficacy, but also demonstrates high safety.

## 3. Materials and Methods

### 3.1. Reagents and Instruments

All chemical agents and solvents, including 1,1,2-Trimethyl-1H-benz[e]indole (compound **1**) and N-Ethyl-3-carbazolecarboxaldehyde (compound **3**), were provided by commercial suppliers (Shanghai Aladdin Biochemical Technology Co., Ltd., Shanghai, China) without further purification. TLC was used to monitor all reactions. The silica gel column was used to purify the reaction products. Moreover, 2′,7′-dichlorodihydrofluorescein (DCFH) and dihydroethidium (DHE) were purchased from Meilun Pharmaceutical (Dalian Meilun Biotechnology Co., Ltd., Dalian, China). Hydroxyphenyl fluorescein (HPF) was purchased from Maclean Reagent (Macklin Biochemical Co., Ltd., Shanghai, China). Singlet oxygen sensor green (SOSG) reagent was purchased from Dalian Meilun Pharmaceutical (Dalian Meilun Biotechnology Co., Ltd., Dalian, China). Furthermore, 2′,7′-Dichlorodihydrofluorescein diacetate (DCFH-DA) was purchased from Sigma-Aldrich (Sigma-Aldrich (Shanghai) Trading Co., Ltd., St. Louis, MO, USA). Fetal bovine serum (FBS) was purchased from Thermo Fisher Scientific (Thermo Fisher Scientific Co., Ltd., Waltham, MA, USA). MTT, DMSO, DAPI, and paraformaldehyde solution (4% PFA) were purchased from Beyotime Biotechnology (Beyotime Biotechnology Co., Ltd., Shanghai, China). Live/dead viability/cytotoxicity kits were purchased from Meilun Pharmaceutical (Dalian Meilun Biotechnology Co., Ltd., Dalian, China).

The target compounds were characterized using ^1^H NMR, ^13^C NMR, and High-Resolution Mass Spectrometry (HRMS). ^1^H NMR and ^13^C NMR were recorded on a Bruker Advance DPX spectrometer from Bruker Corporation (Bruker BioSpin GmbH, Rheinstetten, Germany) at 400 and 100 MHz, respectively. Chemical shifts δ were reported in parts per million (ppm) and coupling constants J in hertz (Hz). HRMS was recorded using an Agilent Technologies LC/MSD TOF from Agilent Technologies (Agilent Technologies Inc., Santa Clara, CA, USA). The UV-Vis absorption spectra were recorded on a spectrometer (Shimadzu Corporation, Kyoto, Japan). The excitation and emission spectra were measured on a SHIMADZU RF-5301PC Fluorescence Spectrometer from Shimadzu Corporation (Shimadzu Corporation, Kyoto, Japan). Tissue sections were obtained through a Leica RM2245 semiautomatic rotary slicer from Leica Microsystems (Leica Camera AG, Wetzlar, Germany). The CLSM images were acquired using a Leica TCS SP8 from Leica Microsystems (Leica Camera AG, Wetzlar, Germany). The laser source we used was LWRPD-200F from Beijing Laserwave Optoelectronics (Beijing Laserwave Optoelectronics Technology Co., Ltd., Beijing, China).

### 3.2. Synthetic Approach

The synthesis processes of compounds **2**, **4**, and **5** were referred to in the previous literature [38,39,40].

#### 3.2.1. Synthetic Method of (E)-2-(2-(9-Ethyl-9H-carbazol-3-yl)vinyl)-1,1,3-trimethyl-1H-benzo[e]indol-3-ium (A1)

First, 9-Ethyl-9H-carbazole-3-carboxaldehyde (223.10 mg, 1.0 mmol) was dissolved with 1,1,2,3-tetramethyl-1H-benzo[e]indol-3-ium (349.03 mg, 1.1 mmol) in anhydrous ethanol in a Schlenk tube of 25 mL, 2 drops of piperidine as the base, and the reaction was carried out at 80 °C for 30 min. After the completion of the reaction, the solvent was spun dry, and the red solid **A1** was purified in a 60% yield using petroleum ether/dichloromethane (1:2, *v*/*v*) as the eluent for column chromatography. ^1^H NMR (400 MHz, DMSO-*d*_6_) *δ* 8.90–8.84 (m, 1H, ArH), 8.60–8.50 (m, 1H, ArH), 8.35–3.32 (m, 1H, ArH), 8.30 (d, *J* = 7.6 Hz, 1H, ArH), 8.17 (q, *J* = 1.9 Hz, 1H, ArH), 8.16–8.11 (m, 2H, 2ArH), 7.84 (ddd, *J* = 9.0, 4.5, 2.5 Hz, 2H, 2ArH), 7.75 (d, *J* = 6.8 Hz, 1H, ArH), 7.72 (s, 1H, ArH), 7.68–7.61 (m, 1H, ArH), 7.57 (td, *J* = 7.6, 1.5 Hz, 1H, ArH), 7.46 (d, *J* = 7.2 Hz, 1H, CH=CH), 7.39–7.27 (m, 1H, CH=CH), 4.54 (q, *J* = 7.0 Hz, 2H, CH_2_), 4.04 (s, 3H, CH_3_), 1.92–1.86 (m, 6H, 2CH_3_), and 1.37 (t, *J* = 7.1 Hz, 3H, CH_3_). ^13^C NMR (101 MHz, CDCl_3_) δ 164.6, 140.7, 129.9, 129.7, 129.0, 128.8, 128.5, 127.2, 126.8, 126.4, 124.1, 123.5, 123.2, 123.1, 121.5, 121.4, 120.8, 120.3, 109.2, 108.7, 108.6, 73.1, 38.0, 29.8, 26.9, and 13.9. ESI-MS (*m*/*z*): calcd for C_31_H_29_N_2_^+^: 429.2325, found 429.2316.

#### 3.2.2. Synthetic Method of (E)-2-(2-(9-Ethyl-6-nitro-9H-carbazol-3-yl)vinyl)-1,1,3-trimethyl-1H-benzo[e]indol-3-ium (A2)

Second, 9-Ethyl-6-nitro-9H-carbazole-3-carboxaldehyde (268.08 mg, 1.0 mmol) was dissolved with 1,1,2,3-tetramethyl-1H-benzo[e]indol-3-ium (349.03 mg, 1.1 mmol) in anhydrous ethanol in a Schlenk tube of 25 mL, and 2 drops of piperidine were used as the base for the reaction, which was carried out at 80 °C for 30 min. After the reaction was complete, the solvent was spun dry and the red solid **A2** was purified via column chromatography using dichloromethane as the eluent in a 65% yield. ^1^H NMR (400 MHz, DMSO-*d*_6_) δ 9.26 (d, *J* = 2.3 Hz, 1H, ArH), 9.15 (d, *J* = 1.6 Hz, 1H, ArH), 8.61 (s, 1H, ArH), 8.43 (dd, *J* = 9.1, 2.3 Hz, 1H, ArH), 8.40–8.35 (m, 1H, ArH), 8.23–8.14 (m, 3H, 3ArH), 7.94 (dd, *J* = 9.0, 7.3 Hz, 2H, 2ArH), 7.86 (d, *J* = 8.7 Hz, 1H, ArH), 7.80–7.73 (m, 2H, 2CH=CH), 7.66 (s, 1H, ArH), 4.62 (q, *J* = 7.3 Hz, 2H, CH_2_), 3.95 (s, 3H, CH_3_), 2.42 (s, 6H, 2CH_3_), and 1.91 (s, 3H, CH_3_). ^13^C NMR (101 MHz, CDCl_3_) δ 193.1, 158.8, 149.8, 143.1, 142.7, 142.3, 142.1, 138.2, 134.1, 131.6, 129.6, 129.5, 128.9, 121.8, 121.4, 121.0, 120.4, 114.5, 113.8, 110.0, 109.9, 32.5, 32.4, 29.7, 24.1, and 23.9. ESI-MS (*m*/*z*): calcd for C_31_H_28_N_3_O_2_^+^: 474.2176, found 474.2166.

#### 3.2.3. Synthetic Method of (E)-2-(2-(6-Amino-9-ethyl-9H-carbazol-3-yl)vinyl)-1,1,3-trimethyl-1H-benzo[e]indol-3-ium (A3)

Third, (E)-2-(2-(9-ethyl-6-nitro-9H-carbazol-3-yl)vinyl)-1,1,3-trimethyl-1H-benzo[e]indol-3-ium (4d) (601.12 mg, 1.0 mmol) was solubilized with ethanol (3 mL), saturated ammonium chloride solution (1 mL) was added, (558.45 mg, 10.0 mmol) iron powder was added, and it was reacted for half an hour. After the reaction was complete, the residual iron powder was washed off with methanol, concentrated under reduced pressure, and purified using dichloromethane: methanol (5:1, *v*/*v*) as the eluent of column chromatography to obtain the purplish-red solid **A3** in a 65% yield. ^1^H NMR (400 MHz, DMSO-*d*_6_) δ 8.44 (d, *J* = 1.7 Hz, 1H, ArH), 8.18 (d, *J* = 8.4 Hz, 1H, ArH), 8.07–8.00 (m, 2H, 2ArH), 7.95 (d, *J* = 8.5 Hz, 1H, ArH), 7.88 (s, 1H, ArH), 7.79 (d, *J* = 8.5 Hz, 1H, ArH), 7.64–7.58 (m, 1H, ArH), 7.54 (d, *J* = 8.6 Hz, 1H, ArH), 7.49 (s, 1H, CH=CH), 7.40–7.31 (m, 3H, 3ArH), 6.87 (s, 1H, CH=CH), 4.94 (s, 2H, NH_2_), 4.36 (q, *J* = 6.8 Hz, 2H, CH_2_), 3.96 (s, 3H, CH_3_), 1.67 (s, 6H, 2CH_3_), and 1.30 (t, *J* = 7.0 Hz, 3H, CH_3_). ^13^C NMR (101 MHz, DMSO-*d_6_*) δ 192.4, 158.6, 149.4, 148.5, 143.9, 140.6, 138.1, 138.0, 135.4, 130.1, 129.8, 128.9, 128.7, 127.3, 126.6, 125.8, 123.9, 122.9, 122.7, 122.3, 120.0, 119.3, 111.7, 110.3, 45.5, 38.0, 37.8, 20.1, and 14.4. ESI-MS (*m*/*z*): calcd for C_31_H_30_N_3_^+^: 444.2434, found 444.2427.

#### 3.2.4. Synthetic Method of (E)-2-(2-(6-Bromo-9-ethyl-9H-carbazol-3-yl)vinyl)-1,1,3-trimethyl-1H-benzo[e]indol-3-ium (A4)

Fourth, 6-Bromo-9-ethyl-9H-carbazole-3-carboxaldehyde (301.01 mg, 1.0 mmol) was dissolved with 1,1,2,3-tetramethyl-1H-benzo[e]indol-3-ium (601.12 mg, 1.1 mmol) in anhydrous ethanol in a 25 mL Schlenk tube, with 2 drops of piperidine as the base, and the reaction was carried out at 80 °C for 30 min. After the reaction was complete, the solvent was spun dry and purified to a dark red solid in a 65% yield using dichloromethane: methanol (5:1, *v*/*v*) as the eluent for column chromatography. ^1^H NMR (400 MHz, DMSO-*d_6_*) δ 8.91–8.83 (m, 1H, ArH), 8.35 (dd, *J* = 8.4, 3.2 Hz, 1H, ArH), 8.30 (d, *J* = 7.6 Hz, 1H, ArH), 8.19–8.10 (m, 3H, 3ArH), 7.84 (ddd, *J* = 8.9, 4.5, 2.2 Hz, 2H, 2ArH), 7.73 (d, *J* = 8.3 Hz, 2H, 2ArH), 7.72–7.70 (m, 2H, 2ArH), 7.60–7.55 (m, 1H, CH=CH), 7.36–7.33 (m, 1H, CH=CH), 4.54 (q, *J* = 7.1 Hz, 2H, CH_2_), 4.04 (s, 3H, CH_3_), 1.92–1.86 (m, 6H, 2CH_3_), and 1.37 (t, *J* = 7.1 Hz, 3H, CH_3_). ^13^C NMR (101 MHz, CDCl_3_) δ 192.9, 158.8, 149.8, 148.3, 142.8, 141.9, 138.1, 134.0, 129.6, 129.5, 129.3, 129.3, 128.1, 123.8, 123.4, 123.2, 122.2, 118.4, 117.0, 110.8, 109.9, 32.5, 32.4, 24.1, 24.1, and 23.9. ESI-MS (*m*/*z*): calcd for C_31_H_28_BrN_2_^+^: 507.1430, found 507.1421.

### 3.3. In Vitro ROS Detection

Fluorescence spectroscopy was used to evaluate the ability and nature of **A1**–**A4** to generate ROS. Firstly, 2′,7′-Dichlorodihydrofluorescein (DCFH) was employed as a universal ROS indicator, while dihydroethidium (DHE) and hydroxyphenyl fluorescein (HPF) served as Type I ROS indicators, and singlet oxygen sensor green (SOSG) reagent was used as a Type II ROS indicator. The total ROS indicator DCFH is oxidized to dichlorofluorescein (DCF) in the presence of ROS in the system, leading to an increase in the fluorescence intensity at 523 nm. DHE, as a superoxide anion indicator, reacts with superoxide anion to generate ethidium, enhancing the fluorescence intensity at 595 nm. HPF, used to detect hydroxyl radicals, exhibits increased fluorescence intensity at 515 nm. The SOSG reagent, as a singlet oxygen indicator, is oxidized to fluorescein upon interaction with singlet oxygen, resulting in enhanced fluorescence intensity at 520 nm. During the experiments, **A1**–**A4** were dissolved in an aqueous solution containing 5% DMSO, with the final concentration adjusted to 10 μM. The concentrations of DCFH, HPF, and DHE were set to 10 μM, while the SOSG reagent was used at 5 μM. At different time points, the samples were irradiated with a 520 nm laser (power density: 100 mW/cm^2^). Subsequently, fluorescence emission spectra were recorded using [Ru(bpy)_3_]^2+^ (RuB) as a positive control for ROS generation. For all indicators (DCFH, DHE, HPF, and SOSG reagent), the excitation wavelength was fixed at 488 nm.

### 3.4. ESR Detection

EPR spectra were recorded using an ESR spectrometer model: A300-10/12 from Bruker (Bruker BioSpin GmbH, Rheinstetten, Germany), with DMPO (5,5-dimethyl-1-pyrroline N-oxide) from Sigma-Aldrich (Sigma-Aldrich (Shanghai) Trading Co., Ltd., St. Louis, MO, USA) as the O_2_^•−^,•OH trapping agent and TEMP (4-amino-2,2,6-tetramethylpiperidine) from Sigma-Aldrich (Sigma-Aldrich (Shanghai) Trading Co., Ltd., St. Louis, MO, USA) as the ^1^O_2_ trapping agent. The free radical signals of **A4** were detected under laser irradiation and non-irradiation conditions. Light source: 520 nm, 100 mW/cm^2^, 10 min.

### 3.5. DFT

Computational DFT calculations were performed using Gaussian09 software (Gaussian, Inc., Wallingford, CT, USA). An initial model was generated using the crystal structure and the resulting data was analyzed using GaussView5 from Gaussian, Inc. (Gaussian, Inc., Wallingford, CT, USA).

### 3.6. Cell Lines and Culture Conditions

Mouse breast adenocarcinoma cell line (4T1), human lung adenocarcinoma cell line (A549), and human colorectal adenocarcinoma cell line (HT29) were purchased from (Shanghai Institute of Cell Biology, Shanghai, China). Cells were cultured in DMEM containing 10% fetal bovine serum and 1% penicillin–streptomycin (100×). The incubation conditions were set at 37 °C, 5% CO_2_, and 95% humidity. Cells were incubated at 37 °C under hypoxic conditions with 2% O_2_ and 5% CO_2_.

### 3.7. Cytotoxicity

Cells (1 × 10^5^ cells) were seeded and cultured under normoxic conditions (21% O_2_) or hypoxic conditions (2% O_2_) for 24 h. Different concentrations of **A1**–**A4** were incubated with the cells in the dark for 24 h. For the light-treated groups, cells were irradiated with a 520 nm laser (100 mW/cm^2^) for 10 min and further cultured for 24 h. Under hypoxic conditions (37 °C, 2% O_2_, and 5% CO_2_), cells were incubated in a tri-gas incubator for 12 h, treated with **A4** (0.125, 0.25, 0.5, 1, and 2 μM) with or without 520 nm laser irradiation (100 mW/cm^2^, 10 min), and then cultured for an additional 24 h. Subsequently, MTT solution (0.5 mg/mL, 20 μL) was added, and cells were incubated at 37 °C for 4 h. The medium was then removed, and 150 μL of DMSO was added to each well to dissolve the formazan crystals. Absorbance at 490 nm was measured using a microplate reader to determine the cell growth inhibition rate.

### 3.8. Staining of Live and Dead Cells

HT-29 (2 × 10^5^ cells/mL) was treated with DMEM containing **A4** (5 μM) for 1 h. Subsequently, the cells were incubated with Calcein-AM (2 μM) and PI (8 μM) for another 30 min. Finally, images were acquired using a Leica TCS SP8 confocal microscope. The excitation wavelength was 488 nm, and the emission wavelengths of Calcein-AM and PI were 500–530 nm and 600–630 nm, respectively.

### 3.9. Intracellular ROS Detection

The HT-29 cells (1 × 10^4^ cells/well) were cultured for 24 h. The cells were then incubated with **A4** (5 μM) under normoxic (21% O_2_) or hypoxic (2% O_2_) conditions for 2 h, followed by co-incubation with DCFH-DA or DHE (10 μM) for 30 min. Hoechst 33342 (10 μM, Beyotime Biotechnology, Shanghai, China) was used for nuclear staining, and imaging was performed using a Leica TCS SP8 confocal fluorescence microscope.

### 3.10. Bacterial Culture

MRSA strains and type identification were provided by the subject of Mr. Yuan Zhenwei of China Pharmaceutical University, and all the above strains were cultured in LB liquid medium (Haibo Biotechnology Co., Ltd., Qingdao, China) in a 37 °C incubator.

### 3.11. In Vitro Antimicrobial Assay

The evaluation of antibacterial activity was carried out using broth microdilution (turbidimetric method), CCK-8 assay, and the plate coating method on MRSA.

Turbidimetric method: Nutrient broth (NB) medium was used to prepare a two-fold dilution of drug-containing suspensions. In a 96-well plate, 100 μL of the diluted bacterial suspension and 100 μL of liposome solution were added to each well, resulting in a final volume of 200 μL per well. After light irradiation, the plate was incubated at 37 °C for 16 h. Turbidity was observed and compared with the control, and the absorbance at 600 nm was measured. OD_600_ represents turbidity, measured at a wavelength in the yellow range. A linear relationship exists between the bacterial concentration (dry weight) and absorbance at 600 nm.

CCK-8 assay: Nutrient broth (NB) medium was used to prepare a two-fold dilution of drug-containing suspensions. In a 96-well plate, 100 μL of the diluted bacterial suspension and 100 μL of liposome solution were added to each well, resulting in a final volume of 200 μL per well. After light irradiation, the plate was incubated at 37 °C for 16 h. Then, 10 μL of CCK-8 solution was added to each well, and absorbance at 450 nm was measured.

Plate coating method for antibacterial activity evaluation: MRSA was selected as the test strain. A 100 μL bacterial suspension (1.0 × 10^5^ CFU/mL) was added to a 96-well plate, with four parallel groups (**A1**, **A2**, **A3**, **A4**) set up to maintain a final volume of 200 μL. Each group was irradiated with a 520 nm laser for 5 min. Subsequently, 100 μL of the treated bacterial suspensions from different groups was spread onto agar plates and incubated at 37 °C for 24 h. Finally, the colonies were counted to determine the synergistic photodynamic antibacterial activity of **A4**.

### 3.12. Bacterial-Level ROS

Positive MRSA bacteria were selected to assess the ROS-generating capacity of bacterial-level **A4**. Three groups were set up in parallel (PBS, **A4**, and **A4** + L). In 1 mL of bacterial suspension (1.0 × 10^8^ CFU/mL), DCFH-DA (ROS commercial fluorescent probe) was added and incubated at 37 °C in an incubator for 30 min. Then, the bacterial suspension was centrifuged (5000 rpm, 3 min) after being treated in different experimental conditions in different groups, rinsed twice with sterile saline (0.9%), and then, the suspension was resuspended in 0.9% saline and added to a laser confocal dish, and the green fluorescence intensity of each group was observed via confocal microscopy to evaluate the ROS production ability at the bacterial level.

### 3.13. Evaluation of Bacterial Membrane Rupture via Scanning Electron Microscopy

MRSA was selected as the test strain. SEM was performed to evaluate the disruption of bacterial membranes by **A4** under 520 nm laser irradiation. Four parallel groups (PBS + L, Van, **A4**, and **A4** + L) were established. Bacterial suspensions with OD_600_ values of 0.5–0.8 were treated with **A4** (1 μM) under different experimental conditions. The samples were then processed as follows: centrifugation, supernatant removal, collection of bacterial pellets, washing three times with 0.9% saline solution, fixation with 2% glutaraldehyde, dehydration, and imaging via SEM to characterize the bacterial membrane morphology.

### 3.14. Construction of an Animal Superficial Infection Model

ICR mice were selected as experimental subjects (18 g, 6–8 weeks), anesthetized with isoflurane(RWD Life Science Co., Ltd., Shenzhen, China), their hair was removed from the surgical area, and a 1 × 1 cm^2^ skin wound was created with a scalpel. The wounds were cleaned with saline and then infected with MRSA bacterial solution (1 × 10^8^ CFU, 200 μL) for 1 day. The superficial bacterial infection model was established on mice. During the experimental period, the wounds were covered with sterile gauze and secured with an elastic bandage, and adequate food and water were given.

### 3.15. Evaluation of Therapeutic Efficacy in Animal Skin Infection Models

After the successful establishment of the animal skin infection model, five parallel groups (0.9% NaCl, 0.9% NaCl +L, 0.4 mg/kg Van, 0.2 mg/kg **A4**, and 0.2 mg/kg **A4** +L) were set up, with 5 mice per group. During treatment, 200 μL of the corresponding drug solution was topically sprayed onto the infected skin area. Light-treated groups received 520 nm laser irradiation (5 min) on the wound region, while non-irradiated groups were kept in the dark. The wound size and healing status of the infected skin were statistically analyzed daily to evaluate therapeutic efficacy under different treatments. On day 8, skin tissues from the wound sites were harvested and fixed with 4% paraformaldehyde. Histopathological sections were prepared and stained with hematoxylin and eosin (H&E) to assess granulation tissue formation and wound healing progression. Masson’s trichrome staining was further performed to evaluate collagen deposition.

### 3.16. In Vivo Antitumor Studies

All animal experiments were approved by the Animal Research and Care Committee of Nantong University (Approval No. S20210925-003) and complied with the National Research Council’s Guide for the Care and Use of Laboratory Animals. Female Balb/c-nude mice (6–8 weeks old, 18–20 g) were purchased from Nanjing Jicui Yaokang Biotechnology Co., Ltd. (Nanjing, China). A 100 μL PBS suspension containing 2 × 10^6^ HT29 cells was subcutaneously injected into the right flank of each mouse. When the tumor volumes reached 80 mm^3^, the mice were randomly divided into four groups (n = 4 per group): PBS, RuB + L, **A4**, and **A4** + L. Drugs were administered via intratumoral injection at a dose of 10 mg/kg. At 2 h post injection, the light-treated groups (RuB + L and **A4** + L) were irradiated with a 520 nm laser (100 mW/cm^2^, 10 min). Treatments were repeated every 3 days for a total of 5 cycles. Next, the tumor volumes as well as body weights of mice in different treatment groups were recorded for 15 days. At the end of the experiment, the mice were euthanized, and the tumors were excised and weighed for antitumor evaluation. The excised tumors and major organs were sectioned and stained with hematoxylin and eosin (H&E) to observe the morphological changes in tumor cells. Masson’s trichrome staining was used to assess collagen degradation. The tumor volume (V) was calculated using the following formula: V = 1/2 × a × b^2^. V represents the tumor volume, a represents the longest diameter of the tumor site, and b represents the diameter perpendicular to a.

### 3.17. H&E Coloring

After the drug effect experiment, Balb/c mouse tumors and major organs such as the heart, liver, spleen, lung, kidney, and so on were dissected and placed in 4% paraformaldehyde for fixation, and then they were subjected to routine operations such as dehydration, transparency, paraffin embedding, and sectioning, etc. Finally, they were stained with H&E using a hematoxylin eosin staining kit (Beyotime Biotechnology Co., Ltd., Shanghai, China) for the relevant histopathological analyses.

### 3.18. Blood Biochemistry Analysis

**A4** was administered to ICR mice at a dose of 5 mg/kg via the tail vein, and blood was obtained 1 day later for relevant blood biochemical analyses: the first category was renal function markers, including creatinine and blood urea nitrogen; the second category was liver function markers, including alanine aminotransferase and aspartate aminotransferase from (Jiancheng Bioengineering Institute, Nanjing, China).

## 4. Conclusions

In this study, we designed and synthesized novel carbazole/benzindole photosensitizers **A1**–**A4**, and evaluated the antimicrobial and antitumor activities of compound **A4** under the synergistic effect of PDT, revealing its dual therapeutic potential. In in vitro antimicrobial assays, **A4** exhibited a concentration-dependent inhibitory effect on MRSA by significantly inducing ROS generation under light conditions, and its antimicrobial activity was comparable to that of vancomycin and superior to that of levofloxacin. Scanning electron microscopy and fluorescence probe experiments further confirmed that **A4** synergistically exerted its bactericidal effect by disrupting the integrity of bacterial membranes and inducing ROS burst. In the MRSA-infected mouse skin wound model, **A4** combined with light treatment not only eliminated the wound bacteria completely, but also accelerated wound healing significantly (the wound area was reduced to 0.03% by day 7), and achieved functional repair by promoting collagen deposition and epidermal regeneration.

In addition, in the HT-29 tumor model, **A4**’s PDT synergistic chemotherapy strategy demonstrated excellent antitumor effects (96% inhibition rate) and did not trigger significant hepatic or renal functional damage or histopathological abnormalities, indicating its good in vivo safety. Comprehensively, **A4** achieved dual precision intervention against drug-resistant bacterial infections and solid tumors by targeting and regulating ROS generation through photodynamic effects. These multifunctional properties provide a theoretical basis for its application in clinical combination therapy, especially in complex cases of drug-resistant bacterial infections combined with tumors. Future studies are needed to further analyze the molecular targets of **A4**, optimize its photosensitivity stability and delivery efficiency, and explore its efficacy in multidrug-resistant pathogens and metastatic tumor models.

## Data Availability

The data presented in this study are available in the article.

## Supplementary Materials

The following supporting information can be downloaded at: https://www.mdpi.com/article/10.3390/molecules30122560/s1. Additional experimental details and methods, including synthetic procedures for compounds **A1**–**A4**, characterization data (^1^H NMR, ^13^C NMR, and ESI-HRMS), and HPLC purity analysis (Figures S12–S27). Photophysical properties: UV-Vis absorption spectra, fluorescence emission spectra (Figures S1–S4), and ESR detection of ROS (Figure S6). In vitro cytotoxicity assays under normoxic/hypoxic conditions (MTT results, Table S1), inhibitory activity of A4 on hypoxic HT29 cells with/without irradiation (Figure S5), live/dead cell staining (Figure S8), and intracellular ROS detection (DCFH-DA and DHE assays, Figure 4). Antibacterial studies: turbidimetric method, CCK-8 assay, plate coating method, bacterial membrane integrity evaluation via SEM (Figure 5n), and bacterial-level ROS generation (Figure S9). In vivo models: MRSA-infected skin wound healing assessment (Figure 6), including histopathological analysis (H&E and Masson staining). Antitumor efficacy in HT-29 xenograft models (Figure 7), tumor volume/weight data, and H&E staining of tumor tissues. Safety evaluations: Blood biochemical analysis (ALT, AST, CREA, UREA) (Figure S11). Histopathology of major organs (heart, liver, spleen, lung, kidney) via H&E staining (Figure S10). Computational studies: HOMO-LUMO energy gaps and spin-state energy differences (ΔE_ST_) for **A4** (Figure S7). Supplementary figures: fluorescence spectra for ROS detection (Figures S1–S4), ESR signals (Figure S6), and confocal microscopy images (Figures S8 and S9).

## Abbreviations

The following abbreviations are used in this manuscript:

PDT	Photodynamic Therapy
ROS	Reactive Oxygen Species
MRSA	Methicillin-resistant *Staphylococcus aureus*
IC_50_	Half-maximal Inhibitory Concentration
HOMO	Highest Occupied Molecular Orbital
LUMO	Lowest Unoccupied Molecular Orbital
ESR	Electron Spin Resonance
CFU	Colony-Forming Units
SEM	Scanning Electron Microscopy
H&E	Hematoxylin and Eosin
ALT	Alanine Aminotransferase
AST	Aspartate Aminotransferase
CREA	Creatinine
UREA	Urea
DCFH-DA	2′,7′-Dichlorodihydrofluorescein Diacetate
CCK-8	Cell Counting Kit-8
RuB	Ruthenium-based photosensitizer
