# Supplementary material for: Novel Type I/II Carbazole/Benzindole Photosensitizers Achieve Chemo-Photodynamic Synergistic Therapy for Suppressing Solid Tumors and Drug-Resistant Bacterial Infections
Source: Molecules. 2025 Jun 12;30(12):2560. doi: 10.3390/molecules30122560 (PMC12195977; doi:10.3390/molecules30122560)
Supplement: Supplementary file 1 [file molecules-30-02560-s001.zip › molecules-3660826-supplementary.pdf]

Material and methods

Details of materials and instruments used, and evaluation protocols of photochemical properties are included in the Supplementary Information.

All chemical agents and solvents were provided from commercial suppliers without further purification. TLC was used to monitor all reactions. The silica gel column was used to purify the reaction products. 2',7'-dichlorodihydrofluorescein (DCFH), and Dihydroethidium (DHE) was purchased from YuanYe Biotechnology Co., Ltd. Hydroxyphenyl fluorescein (HPF) was purchased from MKBio. Singlet Oxygen Sensor Green Reagent (SOSG) was purchased from Meilunbio. 2',7'-Dichlorodihydrofluorescein diacetate (DCFH-DA) was purchased from Sigma-Aldrich. Fetal bovine serum (FBS) was purchased from Gibco (Tulsa, OK). MTT, DMSO, DAPI and Paraformaldehyde solution (4% PFA) were purchased from Beyotime (Shanghai, China). Live/dead viability/cytotoxicity kits were purchased from Meilunbio.

The target compounds were characterized by ^{1}H NMR, ^{13}C NMR, and High Resolution Mass Spectrometry (HRMS). ^{1}H NMR and ^{13}C NMR were recorded on a Bruker Advance DPX spectrometer at 400 and 100 MHz, respectively. Chemical shifts δ are reported in parts per million (ppm) and coupling constants J in hertz (Hz). HRMS was recorded using an Agilent Technologies LC/MSD TOF. The UV-Vis absorption spectra were recorded on a spectrometer (UV1800PC, Jinghua, China). The excitation and emission spectra were measured on a SHIMADZU RF-5301PC Fluorescence Spectrometer. Tissue sections were obtained through a Leica RM2245 semiautomatic rotary slicer (Leica). The CLSM images were acquired using a Leica TCS SP8 (Leica, USA). The laser source we used was LWRPD-200F (Beijing Laserwave).

(E) -2-(2-(9-ethyl-9H-carbazol-3-yl)vinyl)-1,1,3-trimethyl-1H-benzo[e]indol-3-ium (A1)

9-Ethyl-9H-carbazole-3-carboxaldehyde (223.10 mg, 1.0 mmol) was dissolved with 1,1,2,3-tetramethyl-1H-benzo[e]indol-3-ium (349.03 mg, 1.1 mmol) in anhydrous ethanol in a schlenk tube of 25 mL, 2 drops of piperidine as base, and the reaction was carried out at 80 ° C for 30 min. After completion of the reaction, the solvent was spin-dried, and the red solid A1 was purified in 60% yield using petroleum

ether/dichloromethane (1:2, v/v) as eluent for column chromatography. ^{1}H NMR (400 MHz, DMSO-d_6) δ 8.90 – 8.84 (m, 1H, ArH), 8.60 – 8.50 (m, 1H, ArH), 8.35-3.32 (m, 1H, ArH), 8.30 (d, J = 7.6 Hz, 1H, ArH), 8.17 (q, J = 1.9 Hz, 1H, ArH), 8.16 – 8.11 (m, 2H, 2ArH), 7.84 (ddd, J = 9.0, 4.5, 2.5 Hz, 2H, 2ArH), 7.75 (d, J = 6.8 Hz, 1H, ArH), 7.72 (s, 1H, ArH), 7.68 – 7.61 (m, 1H, ArH), 7.57 (td, J = 7.6, 1.5 Hz, 1H, ArH), 7.46 (d, J = 7.2 Hz, 1H, CH=CH), 7.39 – 7.27 (m, 1H, CH=CH), 4.54 (q, J = 7.0 Hz, 2H, CH2), 4.04 (s, 3H, CH3), 1.92 – 1.86 (m, 6H, 2CH3), 1.37 (t, J = 7.1 Hz, 3H, CH3). ^{13}C NMR (101 MHz, $CDCl_3$) δ 164.6, 140.7, 129.9, 129.7, 129.0, 128.8, 128.5, 127.2, 126.8, 126.4, 124.1, 123.5, 123.2, 123.1, 121.5, 121.4, 120.8, 120.3, 109.2, 108.7, 108.6, 73.1, 38.0, 29.8, 26.9, 13.9. ESI-MS (m/z): calcd for $C_{31}H_{29}N_2^+$: 429.2325, found 429.2316.

(E) -2-(2-(9-ethyl-9H-carbazol-3-yl)vinyl)-1,1,3-trimethyl-1H-benzo[e]indol-3-ium(2)

9-Ethyl-9H-carbazole-3-carboxaldehyde (1 mmol) was dissolved with 1,1,2,3-tetramethyl-1H-benzo[e]indol-3-ium (1 mmol) in anhydrous ethanol in a 25 mL schlenk tube, 2 drops of piperidine as base, and the reaction was carried out at 80 ℃ for 30 min. After the reaction was completed, the solvent was spun dry and the red solid 2 was purified in 60 % yield using petroleum ether/dichloromethane (1:2, v/v) as the eluent of column chromatography.

9-Ethyl-6-nitro-9H-carbazole-3-carbaldehyde(2)

9-Ethyl-9H-carbazole-3-carboxaldehyde (223.10 mg, 1.0 mmol) was dissolved in 3 mL of acetic acid, and 1.5 mL of concentrated nitric acid was added dropwise under the condition of an ice bath, and the reaction was carried out for 1 h. Upon completion of the reaction, the solid was filtered and washed with water, and dried to obtain the light yellow product 2 in 75% yield.

(E) -2-(2-(9-ethyl-6-nitro-9H-carbazol-3-yl)vinyl)-1,1,3-trimethyl-1H-benzo[e]indol-3-ium (A2)

9-Ethyl-6-nitro-9H-carbazole-3-carboxaldehyde (268.08 mg, 1.0 mmol) was dissolved with 1,1,2,3-tetramethyl-1H-benzo[e]indol-3-ium (349.03 mg, 1.1 mmol) in anhydrous ethanol in a schlenk tube of 25 mL, and 2 drops of piperidine were used as the base for the reaction, which was carried out at 80 ℃ for 30 min. After the reaction was

completed, the solvent was spun dry and the red solid A2 was purified by column chromatography using dichloromethane as eluent in 65 % yield. ^{1}H NMR (400 MHz, DMSO-d_6) δ 9.26 (d, J = 2.3 Hz, 1H, ArH), 9.15 (d, J = 1.6 Hz, 1H, ArH), 8.61 (s, 1H, ArH), 8.43 (dd, J = 9.1, 2.3 Hz, 1H, ArH), 8.40 – 8.35 (m, 1H, ArH), 8.23 – 8.14 (m, 3H, 3ArH), 7.94 (dd, J = 9.0, 7.3 Hz, 2H, 2ArH), 7.86 (d, J = 8.7 Hz, 1H, ArH), 7.80 – 7.73 (m, 2H, 2CH=CH), 7.66 (s, 1H, ArH), 4.62 (q, J = 7.3 Hz, 2H, CH2), 3.95 (s, 3H, CH3), 2.42 (s, 6H, 2CH3), 1.91 (s, 3H, CH3). ^{13}C NMR (101 MHz, $CDCl_3$) δ 193.1, 158.8, 149.8, 143.1, 142.7, 142.3, 142.1, 138.2, 134.1, 131.6, 129.6, 129.5, 128.9, 121.8, 121.4, 121.0, 120.4, 114.5, 113.8, 110.0, 109.9, 32.5, 32.4, 29.7, 24.1, 23.9. ESI-MS (m/z): calcd for $C_{31}H_{28}N_3O_2^+$: 474.2176, found 474.2166.

(E)-2-(2-(6-amino-9-ethyl-9H-carbazol-3-yl)vinyl)-1,1,3-trimethyl-1H-benzo[e]indol-3-ium (A3)

(E) -2-(2-(9-ethyl-6-nitro-9H-carbazol-3-yl)vinyl)-1,1,3-trimethyl-1H-benzo[e]indol-3-ium (4d) (601.12 mg, 1.0 mmol) was solubilized with ethanol (3 mL), saturated ammonium chloride solution (1 mL) was added, add (558.45 mg, 10.0 mmol) iron powder and react for half an hour. After the reaction was completed, the residual iron powder was washed off with methanol, concentrated under reduced pressure, and purified by using dichloromethane : methanol (5:1, v/v) as the eluent of column chromatography to obtain the purplish-red solid A3 in 65 % yield. ^{1}H NMR (400 MHz, DMSO-d_6) δ 8.44 (d, J = 1.7 Hz, 1H, ArH), 8.18 (d, J = 8.4 Hz, 1H, ArH), 8.07 – 8.00 (m, 2H, 2ArH), 7.95 (d, J = 8.5 Hz, 1H, ArH), 7.88 (s, 1H, ArH), 7.79 (d, J = 8.5 Hz, 1H, ArH), 7.64 – 7.58 (m, 1H, ArH), 7.54 (d, J = 8.6 Hz, 1H, ArH), 7.49 (s, 1H, CH=CH), 7.40 – 7.31 (m, 3H, 3ArH), 6.87 (s, 1H, CH=CH), 4.94 (s, 2H, NH2), 4.36 (q, J = 6.8 Hz, 2H, CH2), 3.96 (s, 3H, CH3), 1.67 (s, 6H, 2CH3), 1.30 (t, J = 7.0 Hz, 3H, CH3). ^{13}H NMR (101 MHz, DMSO-d_6) δ 192.4, 158.6, 149.4, 148.5, 143.9, 140.6, 138.1, 138.0, 135.4, 130.1, 129.8, 128.9, 128.7, 127.3, 126.6, 125.8, 123.9, 122.9, 122.7, 122.3, 120.0, 119.3, 111.7, 110.3, 45.5, 38.0, 37.8, 20.1, 14.4. ESI-MS (m/z): calcd for $C_{31}H_{30}N_3^+$: 444.2434, found 444.2427.

6-Bromo-9-ethyl-9H-carbazole-3-carbaldehyde(3)

9-Ethyl-9H-carbazole-3-carboxaldehyde (223.10 mg, 1.0 mmol) was dissolved in anhydrous THF, NBS (195.78 mg, 1.1 mmol) was added, and the reaction was carried out at 65 °C for 1 h. Upon completion of the reaction, the solid was withdrawn, and

dried to a white solid Compound 3 in 75% yield.

(E)-2-(2-(6-bromo-9-ethyl-9H-carbazol-3-yl)vinyl)-1,1,3-trimethyl-1H-benzo[e]indol-3-ium (A4)

6-Bromo-9-ethyl-9H-carbazole-3-carboxaldehyde (301.01 mg, 1.0 mmol) was dissolved with 1,1,2,3-tetramethyl-1H-benzo[e]indol-3-ium (601.12 mg, 1.1 mmol) in anhydrous ethanol in a 25 mL schlenk tube, 2 drops of piperidine as base, and the reaction was carried out at 80 ℃ for 30 min. After the reaction was completed, the solvent was spun dry and purified to a dark red solid in 65 % yield using dichloromethane : methanol (5:1, v/v) as eluent for column chromatography. ^{1}H NMR (400 MHz, DMSO-d_6) δ 8.91 – 8.83 (m, 1H, ArH), 8.35 (dd, J = 8.4, 3.2 Hz, 1H, ArH), 8.30 (d, J = 7.6 Hz, 1H, ArH), 8.19 – 8.10 (m, 3H, 3ArH), 7.84 (ddd, J = 8.9, 4.5, 2.2 Hz, 2H, 2ArH), 7.73 (d, J = 8.3 Hz, 2H, 2ArH), 7.72-7.70 (m, 2H, 2ArH), 7.60 – 7.55 (m, 1H, CH=CH), 7.36-7.33 (m, 1H, CH=CH), 4.54 (q, J = 7.1 Hz, 2H, CH2), 4.04 (s, 3H, CH3), 1.92 – 1.86 (m, 6H, 2CH3), 1.37 (t, J = 7.1 Hz, 3H, CH3). ^{13}C NMR (101 MHz, $CDCl_3$) δ 192.9, 158.8, 149.8, 148.3, 142.8, 141.9, 138.1, 134.0, 129.6, 129.5, 129.3, 129.3, 128.1, 123.8, 123.4, 123.2, 122.2, 118.4, 117.0, 110.8, 109.9, 32.5, 32.4, 24.1, 24.1, 23.9. ESI-MS (m/z): calcd for $C_{31}H_{28}BrN_2^+$: 507.1430, found 507.1421.

In vitro ROS detection

Fluorescence spectroscopy was used to evaluate the ability and nature of A1-A4 to generate reactive oxygen species (ROS). 2',7'-Dichlorodihydrofluorescein (DCFH) was employed as a universal ROS indicator, while dihydroethidium (DHE) and hydroxyphenyl fluorescein (HPF) served as type-I ROS indicators, and singlet oxygen sensor green (SOSG) was used as a type-II ROS indicator. The total ROS indicator DCFH is oxidized to dichlorofluorescein (DCF) in the presence of ROS in the system, leading to an increase in fluorescence intensity at 523 nm. DHE, as a superoxide anion indicator, reacts with superoxide anion to generate ethidium, enhancing fluorescence intensity at 595 nm. HPF, used to detect hydroxyl radicals, exhibits increased fluorescence intensity at 515 nm. SOSG, as a singlet oxygen indicator, is oxidized to fluorescein upon interaction with singlet oxygen, resulting in enhanced fluorescence intensity at 520 nm. During the experiments, A1-A4 were dissolved in an aqueous solution containing 5% DMSO, with the final concentration adjusted to 10 μM. The concentrations of DCFH, HPF, and DHE were set to 10 μM, while SOSG was used at

5 μM. At different time points, the samples were irradiated with a 520 nm laser (power density: 100 mW/cm^2). Subsequently, fluorescence emission spectra were recorded using $[Ru(bpy)_3]^{2+}$ (RuB) as a positive control for ROS generation. For all indicators (DCFH, DHE, HPF, SOSG), the excitation wavelength was fixed at 488 nm.

ESR detection

EPR spectra were recorded using an ESR spectrometer (Bruker, Germany; model: A300-10/12), with DMPO (5,5-dimethyl-1-pyrroline N-oxide) as the ·O2⁻,·OH trapping agent and TEMP (4-amino-2,2,6-tetramethylpiperidine) as the 1O2 trapping agent. The free radical signals of A4 were detected under laser irradiation and non-irradiation conditions. light source: 520 nm, 100 mW/cm2, 10 min

DFT

Computational DFT calculations were performed using Gaussian09 software. An initial model was generated using the crystal structure and the resulting data was analyzed using GaussView5.

Cell lines and culture conditions

Mouse breast adenocarcinoma cell line (4T1), human lung adenocarcinoma cell line (A549) and human colorectal adenocarcinoma cell line (HT29) were purchased from Shanghai Institute of Cell Biology. Cells were cultured in DMEM containing 10% fetal bovine serum and 1% penicillin-streptomycin (100X). Incubation conditions were set at 37 °C, 5% CO_2 and 95% humidity. Cells were incubated at 37 °C under hypoxic conditions with 2% O_2 and 5% CO_2.

Cytotoxicity

Cells (1 × 10^5 cells) were seeded and cultured under normoxic conditions (21% O_2) or hypoxic conditions (2% O_2) for 24 h. Different concentrations of A1-A4 were incubated with the cells in the dark for 24 h. For the light-treated groups, cells were irradiated with a 520 nm laser (100 mW/cm^2) for 10 min and further cultured for 24 h. Under hypoxic conditions (37 °C, 2% O_2, 5% CO_2), cells were incubated in a tri-gas incubator for 12 h, treated with A4 (0.125, 0.25, 0.5, 1, and 2 μM) with or without 520 nm laser irradiation (100 mW/cm^2, 10 min), and then cultured for an additional 24 h.

Subsequently, MTT solution (0.5 mg/mL, 20 μL) was added, and cells were incubated at 37 °C for 4 h. The medium was then removed, and 150 μL of DMSO was added to each well to dissolve the formazan crystals. Absorbance at 490 nm was measured using a microplate reader to determine the cell growth inhibition rate.

Live and dead

HT-29 (2 × 105 cells/ml) was treated with DMEM medium containing A4 (5 μM) for 1 h. Subsequently, the cells were incubated with Calcein-AM (2 μM) and PI (8 μM) for another 30 min. Finally, images were acquired using a Leica TCS SP8 confocal microscope. The excitation wavelength was 488 nm, and the emission wavelengths of Calcein-AM and PI were 500-530 nm and 600-630 nm, respectively.

Intracellular ROS detection

HT-29 cells (1×10^4 cells/well) were cultured for 24 h. The cells were then incubated with A4 (5 μM) under normoxic (21% O_2) or hypoxic (2% O_2) conditions for 2 h, followed by co-incubation with DCFH-DA or DHE (10 μM) for 30 min. Hoechst 33342 (10 μM) was used for nuclear staining, and imaging was performed using a Leica TCS SP8 confocal fluorescence microscope.

Bacterial culture

Methicillin-resistant Staphylococcus aureus (MRSA) strains and type identification were provided by the subject of Mr. Yuan Zhenwei of China Pharmaceutical University, and all the above strains were cultured in LB liquid medium at 37°C incubator.

In vitro antimicrobial assay

Evaluation of antibacterial activity using broth microdilution (turbidimetric method), CCK-8 assay, and plate coating method on MRSA

Turbidimetric method: Nutrient broth (NB) medium was used to prepare a two-fold dilution of drug-containing suspensions. In a 96-well plate, 100 μL of the diluted bacterial suspension and 100 μL of liposome solution were added to each well, resulting in a final volume of 200 μL per well. After light irradiation, the plate was incubated at 37 °C for 16 h. Turbidity was observed and compared with the control, and the absorbance at 600 nm was measured. OD_{600} represents turbidity, measured at a wavelength in the yellow range. A linear relationship exists between bacterial concentration (dry weight) and absorbance at 600 nm.

CCK-8 assay: Nutrient broth (NB) medium was used to prepare a two-fold dilution of drug-containing suspensions. In a 96-well plate, 100 μL of the diluted bacterial suspension and 100 μL of liposome solution were added to each well, resulting in a

final volume of 200 μL per well. After light irradiation, the plate was incubated at 37 °C for 16 h. Then, 10 μL of CCK-8 solution was added to each well, and absorbance at 450 nm was measured.

Plate coating method for antibacterial activity evaluation: MRSA was selected as the test strain. A 100 μL bacterial suspension (1.0×10^5 CFU/mL) was added to a 96-well plate, with four parallel groups (A1, A2, A3, A4) set up to maintain a final volume of 200 μL. Each group was irradiated with a 520 nm laser for 5 min. Subsequently, 100 μL of the treated bacterial suspensions from different groups was spread onto agar plates and incubated at 37 °C for 24 h. Finally, colonies were counted to determine the synergistic photodynamic antibacterial activity of A4.

Bacterial level ROS

Positive bacteria methicillin-resistant Staphylococcus aureus (MRSA) were selected to assess the ROS-generating capacity of bacterial level A4. Three groups were set up in parallel (PBS, A4, A4+L). In 1 ml of bacterial suspension (1.0×108 CFU/mL), DCFH-DA (ROS commercial fluorescent probe) was added and incubated at 37°C in an incubator for 30 min, and then the bacterial suspension was centrifuged (5000 rpm, 3 min) after being treated with different experimental conditions in different groups, rinsed twice with sterile saline (0.9%), and then the suspension was resuspended in 0.9% saline and added to a laser confocal dish, and the green fluorescence intensity of each group was observed by confocal microscopy to evaluate the ROS production ability at the bacterial level.

Evaluation of bacterial membrane rupture by scanning electron microscopy

MRSA was selected as the test strain. SEM was performed to evaluate the disruption of bacterial membranes by A4 under 520 nm laser irradiation. Four parallel groups (PBS+L, Van, A4, A4+L) were established. Bacterial suspensions with OD_{600} values of 0.5-0.8 were treated with A4 (1 μM) under different experimental conditions. The samples were then processed as follows: centrifugation, supernatant removal, collection of bacterial pellets, washing three times with 0.9% saline solution, fixation with 2% glutaraldehyde, dehydration, and imaging via SEM to characterize bacterial membrane morphology.

Construction of an animal superficial infection model

ICR mice were selected as experimental subjects (18 g, 6-8 weeks), anesthetized with isoflurane, hair was removed from the surgical area, and a 1×1 cm^2 skin wound was created with a scalpel. The wounds were cleaned with saline and then infected with MRSA bacterial solution (1×108 CFU, 200 μL) for 1 day. Superficial bacterial infection model was established on mice. During the experimental period, the wounds were covered with sterile gauze and secured with an elastic bandage, and adequate food and water were given.

Evaluation of therapeutic efficacy in animal skin infection models

After successful establishment of the animal skin infection model, five parallel groups (0.9% NaCl, 0.9% NaCl +L, 0.4 mg/kg Van, 0.2 mg/kg A4, 0.2 mg/kg A4 +L) were set up, with 5 mice per group. During treatment, 200 μL of the corresponding drug solution was topically sprayed onto the infected skin area. Light-treated groups received 520 nm laser irradiation (5 min) on the wound region, while non-irradiated groups were kept in the dark. The wound size and healing status of the infected skin were statistically analyzed daily to evaluate therapeutic efficacy under different treatments. On day 8, skin tissues from the wound sites were harvested and fixed with 4% paraformaldehyde. Histopathological sections were prepared and stained with hematoxylin and eosin (H&E) to assess granulation tissue formation and wound healing progression. Masson's trichrome staining was further performed to evaluate collagen deposition.

In vivo anti-tumor studies

All animal experiments were approved by the Animal Research and Care Committee of Nantong University (Approval No. S20210925-003) and complied with the National Research Council's Guide for the Care and Use of Laboratory Animals. Female Balb/c-nude mice (6–8 weeks old, 18–20 g) were purchased from Nanjing Jicui Yaokang Biotechnology Co., Ltd. A 100 μL PBS suspension containing 2×10^6 HT29 cells was subcutaneously injected into the right flank of each mouse. When tumor volumes reached 80 mm^3, the mice were randomly divided into four groups (n = 4 per group): PBS, RuB+L, **A4**, and **A4**+L. Drugs were administered via intratumoral injection at a dose of 10 mg/kg. At 2 hours post-injection, the light-treated groups (RuB+L and A4+L) were irradiated with a 520 nm laser (100 mW/cm^2, 10 min). Treatments were repeated every 3 days for a total of 5 cycles. Next, tumor volumes as well as body weights of mice in different treatment groups were recorded for 15 days. At the end of the experiment, mice were euthanized, and tumors were excised and weighed for antitumor evaluation. Excised tumors and major organs were sectioned and stained with hematoxylin and eosin (H&E) to observe morphological changes in tumor cells. Masson's trichrome staining was used to assess collagen degradation. Tumor volume (V) was calculated using the formula: V = 1/2×a×**A3**, V represents the tumor volume, a represents the longest diameter of the tumor site, and b represents the diameter perpendicular to a.

H&E coloring

After the drug effect experiment, Balb/c mouse tumors and major organs such as heart, liver, spleen, lung, kidney and so on were dissected and placed in 4% paraformaldehyde for fixation respectively, and then they were subjected to routine operations such as dehydration, transparency, paraffin embedding, sectioning, etc., and finally they were stained with H&E using hematoxylin eosin staining kit for the relevant histopathological analyses.

Blood biochemistry analysis

A4 was administered to ICR mice at a dose of 5 mg/kg via tail vein, and blood was

obtained 1 day later for relevant blood biochemical analyses: the first category was renal function markers, including creatinine and blood urea nitrogen; the second category was liver function markers, including alanine aminotransferase and aspartate aminotransferase.

Statistical analysis was performed using GraphPad Prism 8 software. In order to compare two groups of data for a significant relationship, a t-test was used. For more than two groups of data, a two-way analysis of variance (ANOVA) was used, *$^*P<0.05$, $^{**}P<0.01$, $^{***}P<0.001$, $^{****}P<0.001$*。

Table S1. IC_{50} values in the dark or under irradiation of complexes **A1,A2,A3,A4** toward three cancer cell lines.

Compd.	Conditions		*In vitro* antiproliferative activity (IC_{50}, μM)		
			HT29	A549	4T1
RuB	normoxia[a]	Dark	> 100	> 100	> 100
		Light	14.90 ± 1.70	27.90 ± 1.30	95.62 ± 16.16
**A1**	normoxia[a]	Dark	6.8±1.20	2.94±0.06	6.87±0.05
		Light	3.26±0.07	1.26±0.13	3.15±0.13
**A2**	normoxia[a]	Dark	9.79±0.57	6.29±0.13	11.41±0.08
		Light	6.12±0.84	2.14±0.02	3.05±0.15
**A3**	normoxia	Dark	14.17±1.35	48.54±6.36	20.32±1.95
		Light	2.24±0.02	4.12±0.06	2.45 ± 0.04
**A4**	normoxia	Dark	9.47±2.76	1.92±0.16	5.70±0.39
		Light	1.10±0.02	0.41±0.01	1.68±0.13
	hypoxia[b]	Dark	9.47±1.33	NS	NS
		Light	0.89±0.02	NS	NS

[a]Cytotoxicity of the tested compound incubated for 24 h in normoxia (21% O_2). [b]Cytotoxicity of the tested compound incubated for 24 h in hypoxia (2% O_2). Irradiation: 520 nm, 100 mW/cm^2, 10 min.

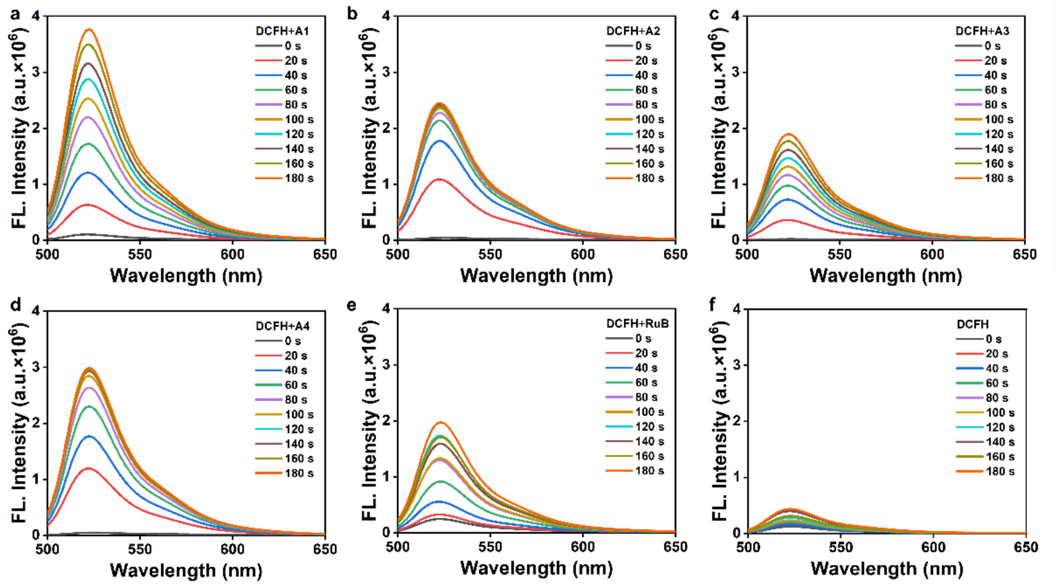

Figure S1. FL spectra of DCFH (10 μM) in the presence of (a) DCFH +**A1**, (b), DCFH +**A2** (c) DCFH +**A3**, (d) DCFH +**A4**, (e) DCFH +RuB**,** (f) DCFH after exposure to 520 nm laser irradiation with different time in mixture solvent DMSO/deionized water solution (v/v = 5: 95). (λ_{ex}=488nm). Concentration: 10 × 10^{-6} M, 10 × 10^{-6} M (DCFH), light irradiation (50 mW/cm^2).

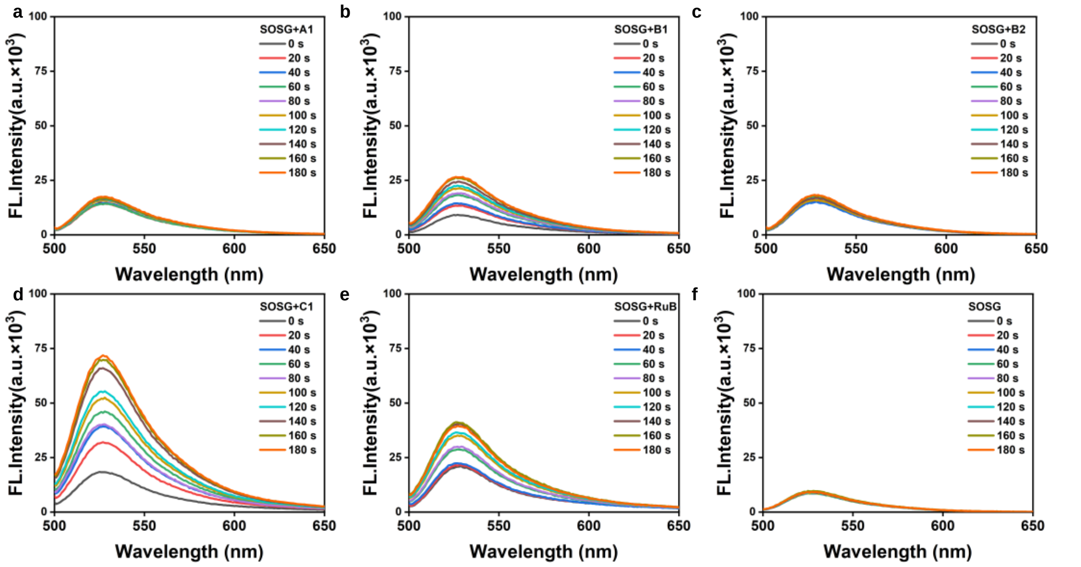

Figure S2. FL spectra of SOSG (5 μM) in the presence of (a) SOSG+**A1**, (b), SOSG +**A2** (c) SOSG +**A3**, (d) SOSG +**A4**, (e) SOSG +RuB**,** (f) SOSG after exposure to 520 nm laser irradiation with different time in mixture solvent DMSO/deionized water solution (v/v = 5: 95). (λ_{ex}=488nm). Concentration: 5 × 10^{-6} M, 5 × 10^{-6} M (SOSG),

light irradiation (50 mW/cm^2).

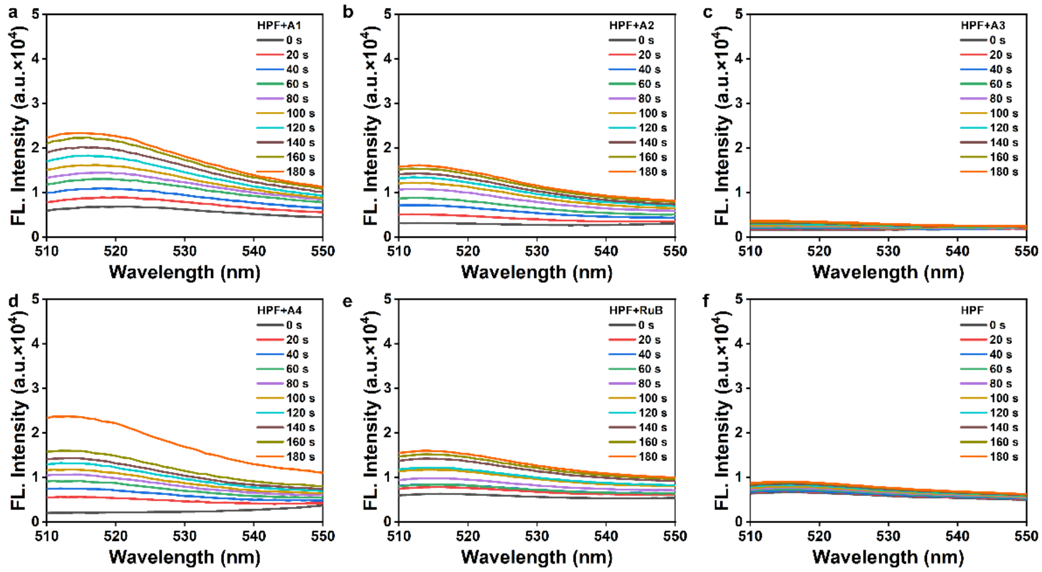

Figure S3. FL spectra of HPF (10 μM) in the presence of (a) HPF+**A1**, (b), HPF+**A2** (c) HPF+**A3**, (d) HPF+**A4**, (e) HPF+RuB**,** (f) HPF after exposure to 520 nm laser irradiation with different time in mixture solvent DMSO/deionized water solution (v/v = 5: 95). (λ_{ex}=488nm). Concentration: 10 × 10^{-6} M, 10 × 10^{-6} M (HPF), light irradiation (50 mW/cm^2).

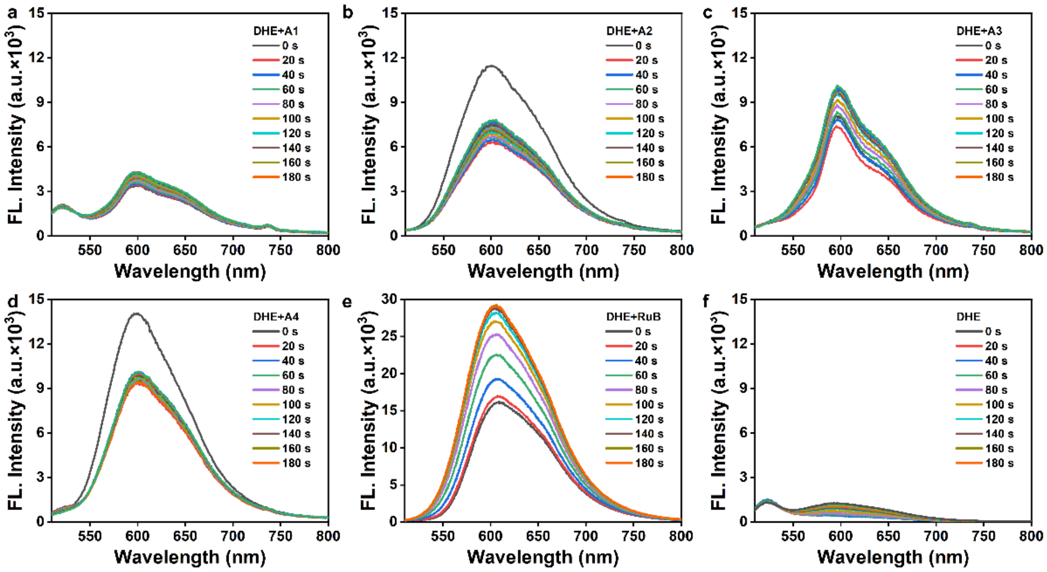

Figure S4. FL spectra of DHE (10 μM) in the presence of (a) DHE+**A1**, (b), DHE +**A2** (c) DHE +**A3**, (d) DHE +**A4**, (e) DHE +RuB**,** (f) DHE after exposure to 520 nm laser irradiation with different time in mixture solvent DMSO/deionized water solution (v/v = 5: 95). (λ_{ex}=488nm). Concentration: 10 × 10^{-6} M, 10 × 10^{-6} M (DHE), light irradiation (50 mW/cm^2).

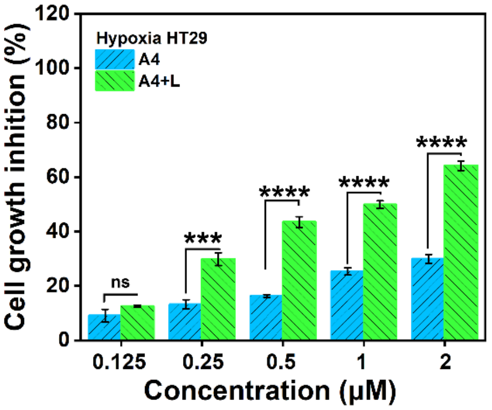

Figure S5. Inhibitory activities of **A4** on hypoxic HT29 cell with/without irradiation. L: light. Light source: 520 nm, 100 mW/cm^2, 10 min, Mean ± SD, n = 3, ****P < 0.001, ****P < 0.0001.*

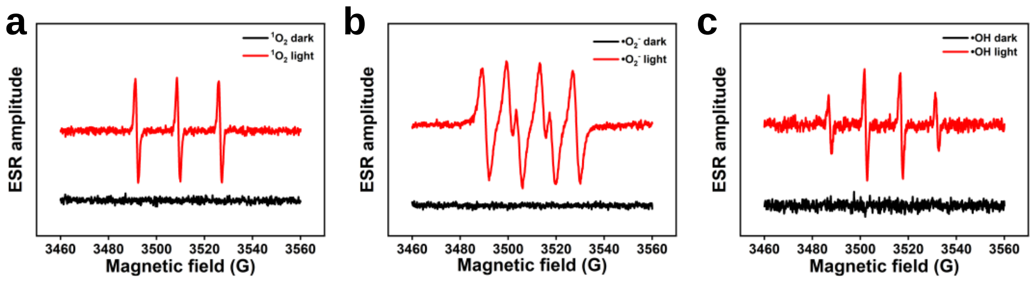

Figure S6. ESR signals of TEMP reacting with (a) $^1O_2$, and DMPO reacting with (b) $\bullet O_2^-$, (c) •OH, and in the presence of **A4** before and after light irradiation. (Light source: 520 nm, 100 mW/cm^2).

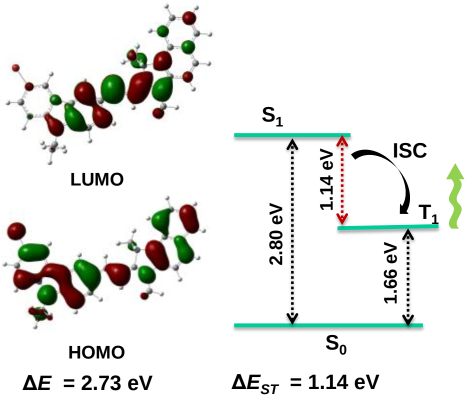

Figure S7. Calculated HOMO, LUMO, $\Delta E_{(LUMO\text{-}HOMO)}$, and ΔE_{ST} for **A4** from TD-DFT （Gaussian/ TD-DFT/6-31G(d).

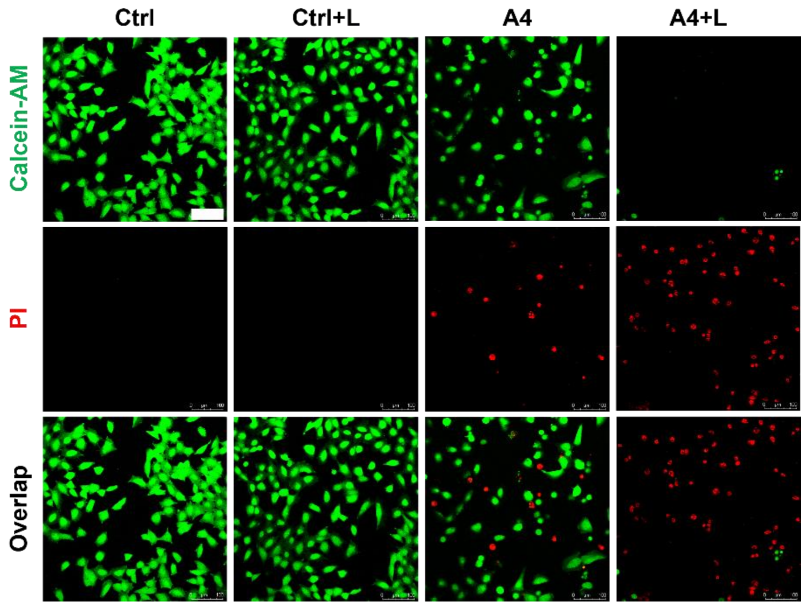

Figure S8. Fluorescence microscopic imaging of HT29 cells in different groups after treatment with Calcein-AM (live cell marker) and propidium iodide (PI) (dead cell marker). (Light source: 520 nm, 100 mW/cm^2, 10 min) (Scale bar = 100 μm).

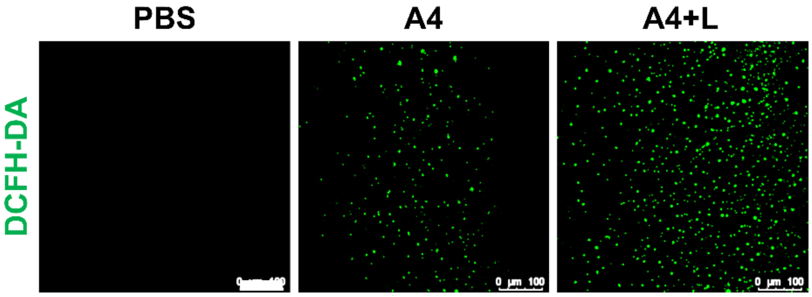

Figure S9. Fluorescence microscopic imaging of MRSA in different groups after treatment with DCFH-DA. (Light source: 520 nm, 100 mW/cm^2, 10 min) (Scale bar = 100 μm).

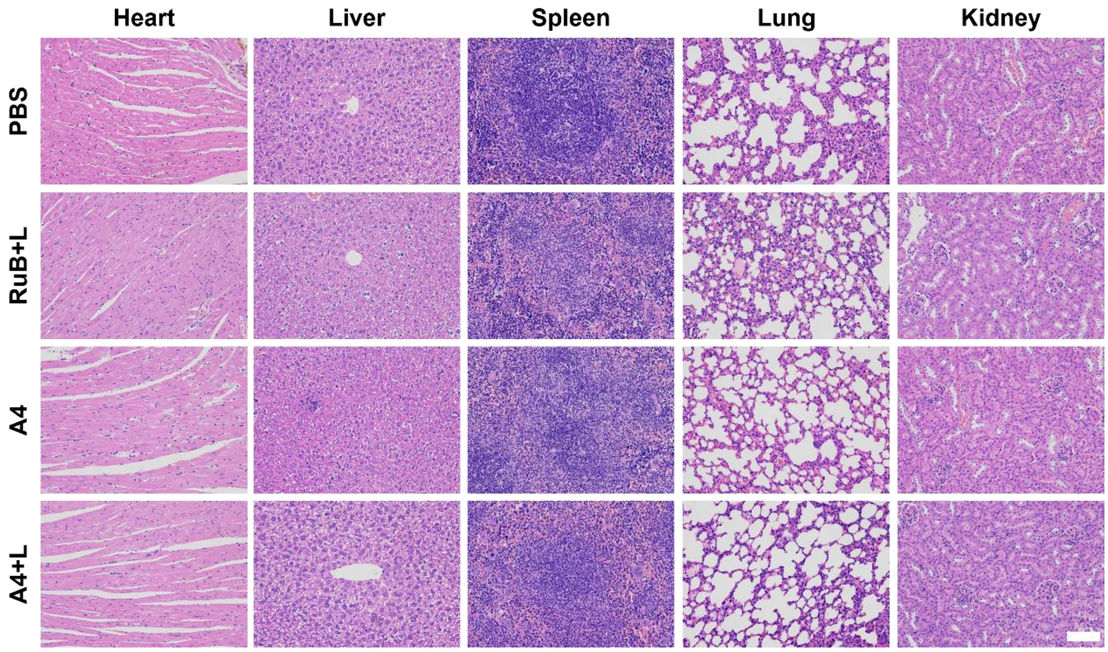

Figure S10. H&E staining of the heart, kidney, spleen, and liver after treatments with PBS, RuB, **A4** and **A4**+L (Scale bar = 50 μm).

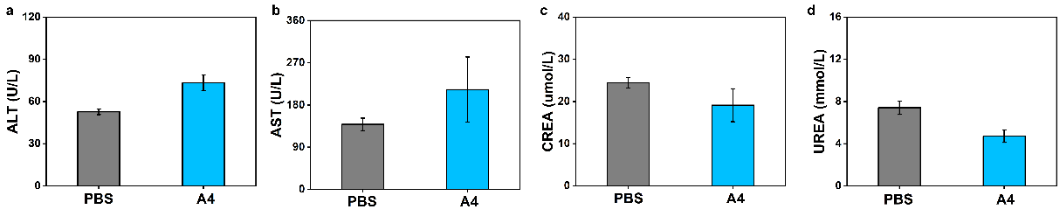

Figure S11. The level of hepatic or renal function markers (ALT, AST, CREA, and BUN) in the blood of mice after treatment with PBS, and **A4**.

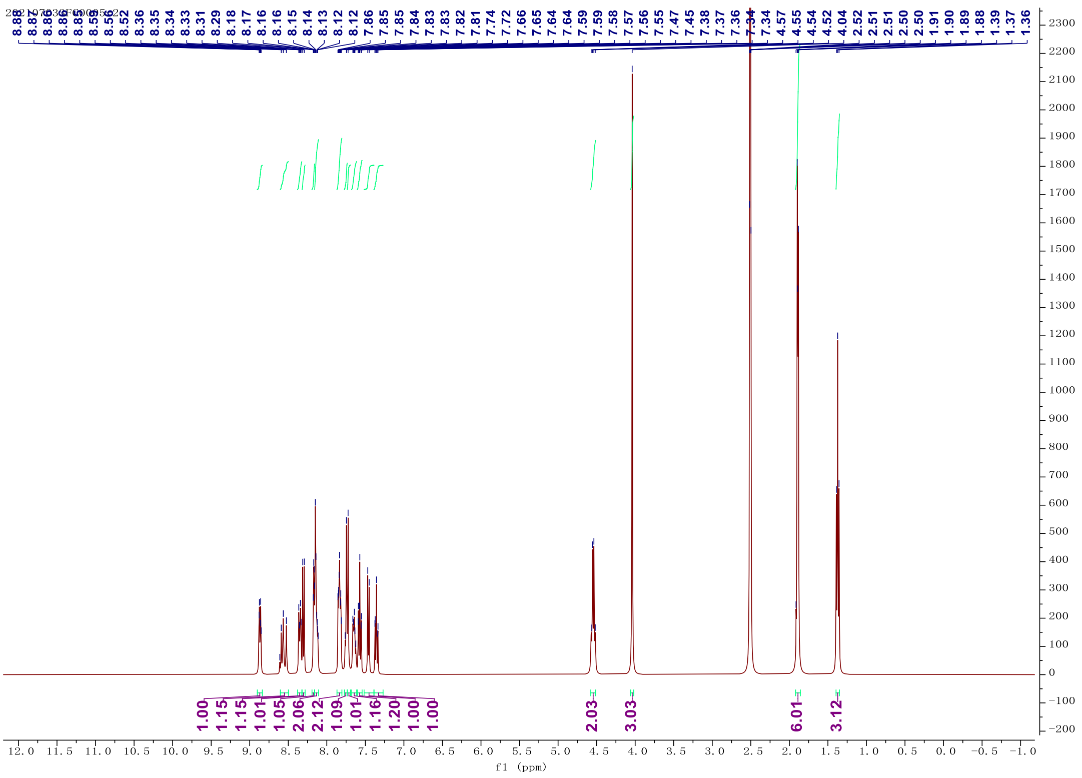

Figure S12. ^{1}H-NMR spectra of compound **A1** in DMSO-d_6.

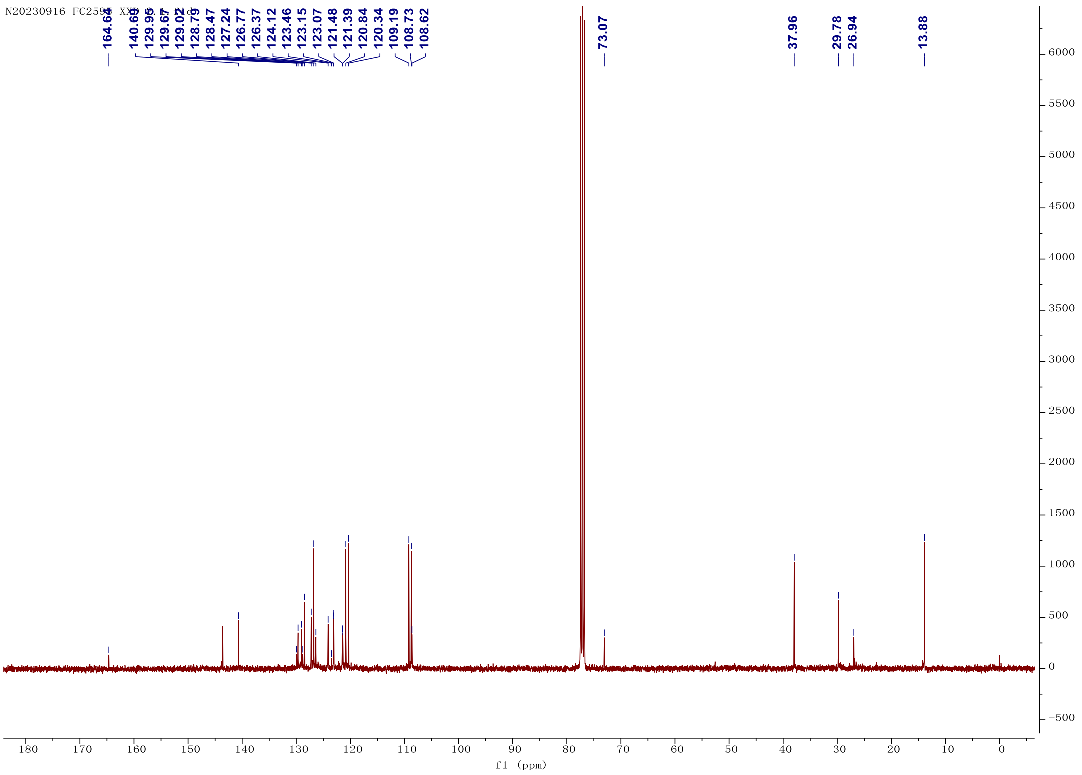

Figure S13. ^{13}C-NMR spectra of compound **A1** in $CDCl_3$.

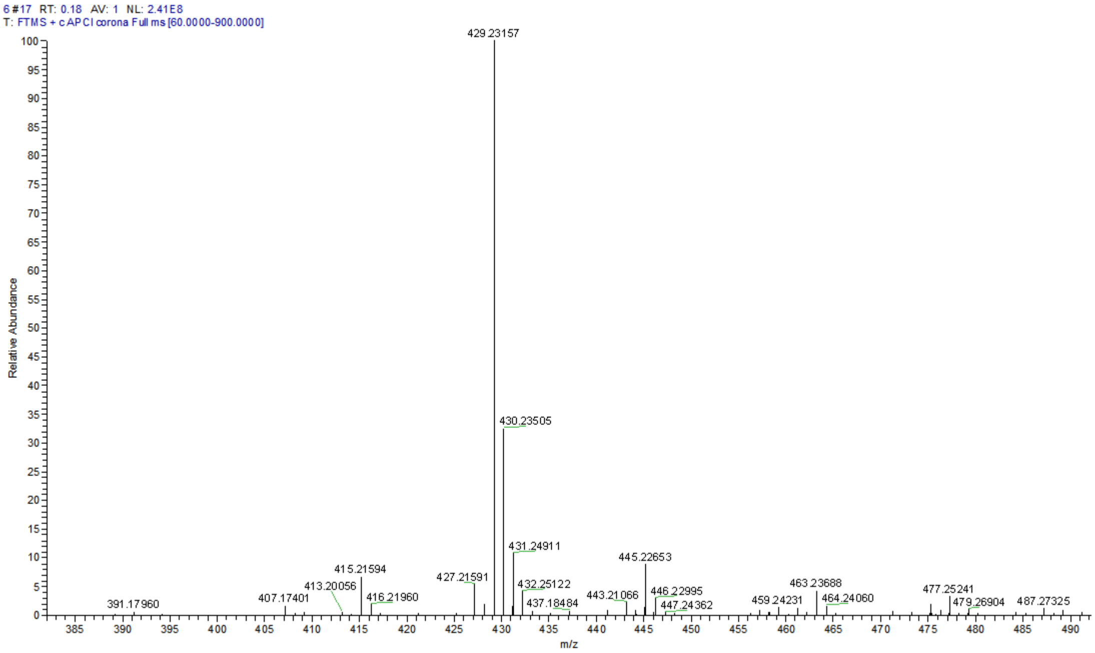

Figure S14. ESI-MS of **A1**.

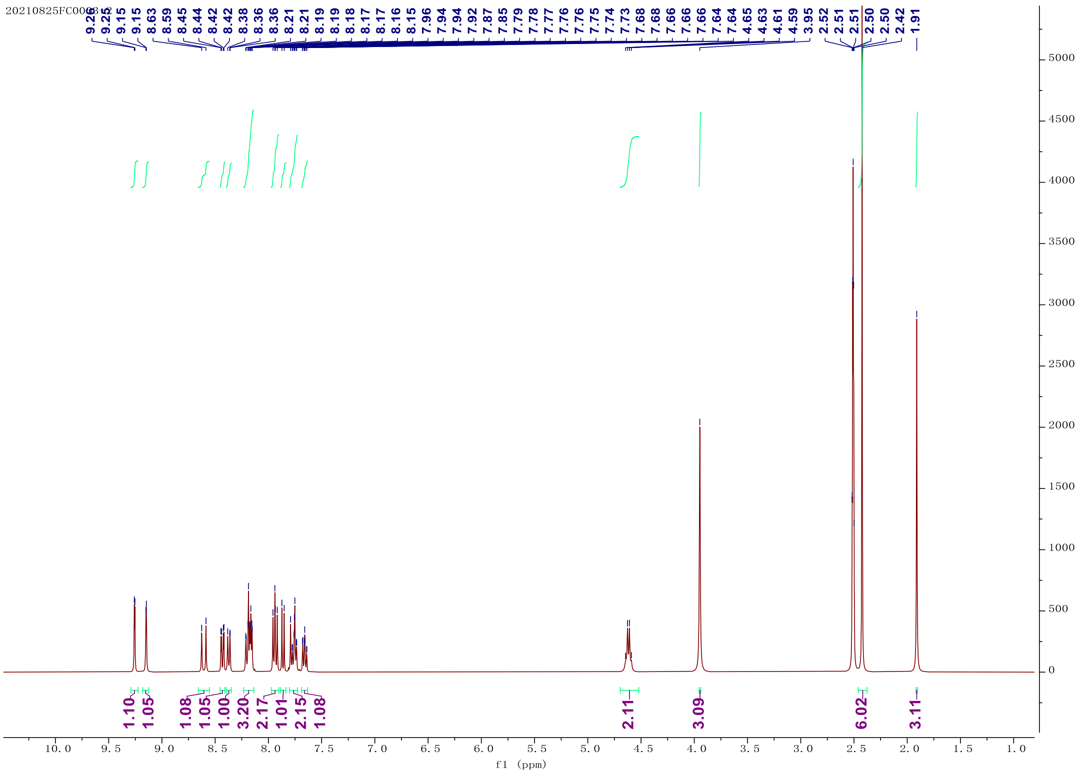

Figure S15. ^{1}H-NMR spectra of compound **A2** in DMSO-d_6.

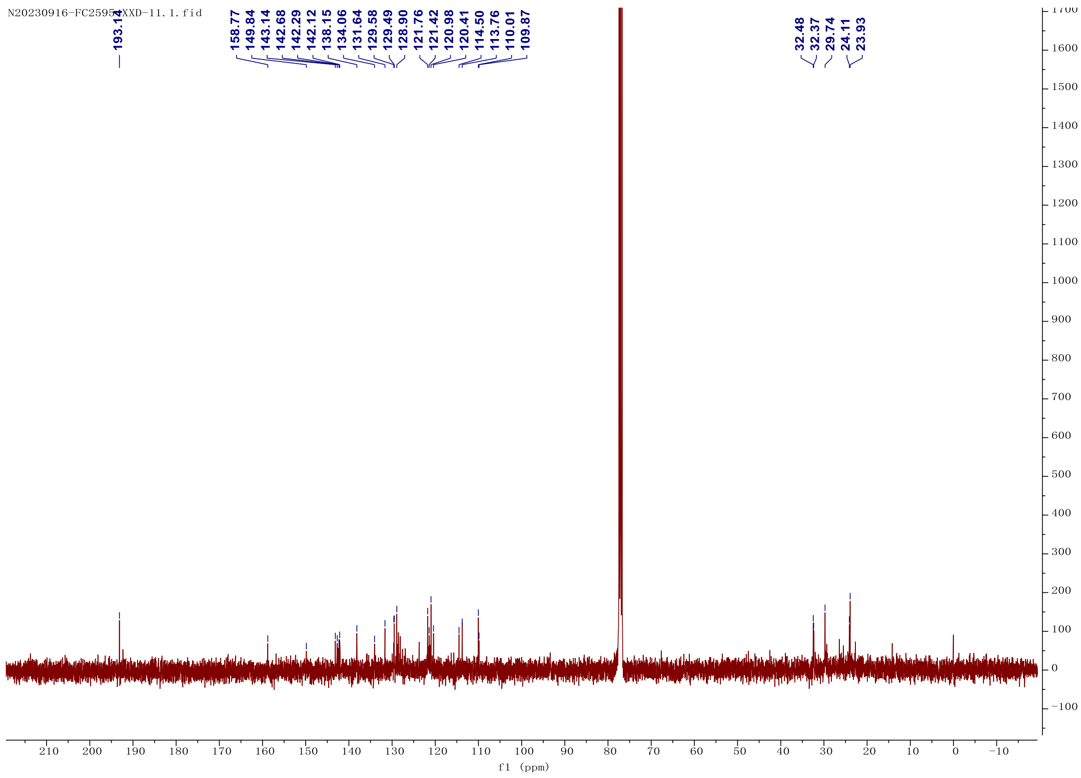

Figure S16. ^{13}C-NMR spectra of compound **A2** in $CDCl_3$.

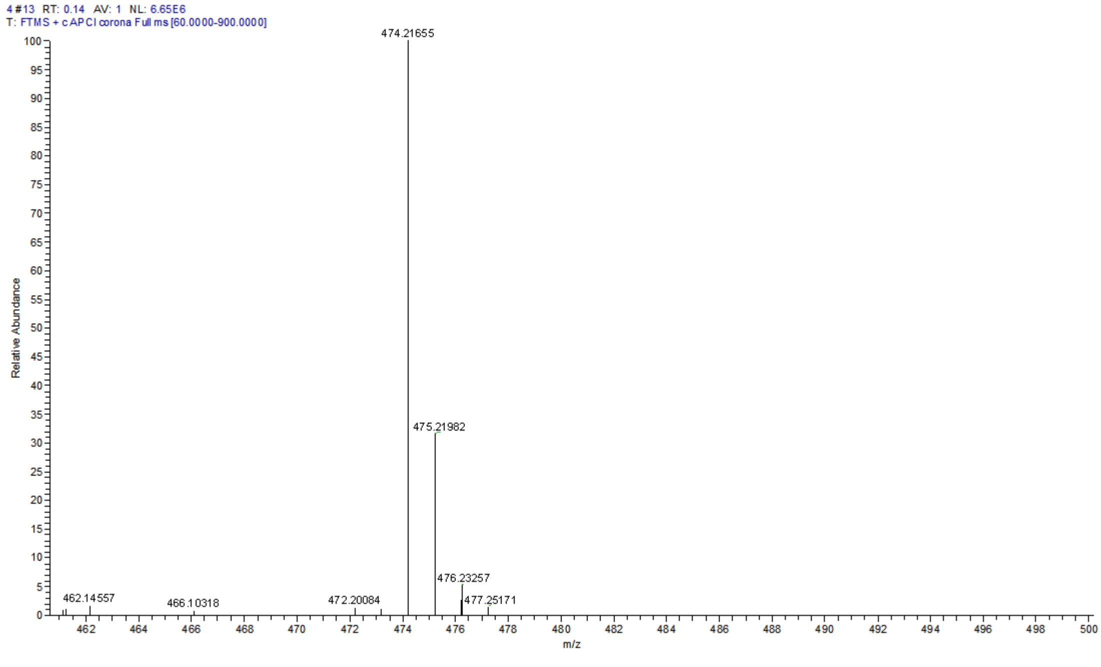

Figure S17. ESI-MS of **A2.**

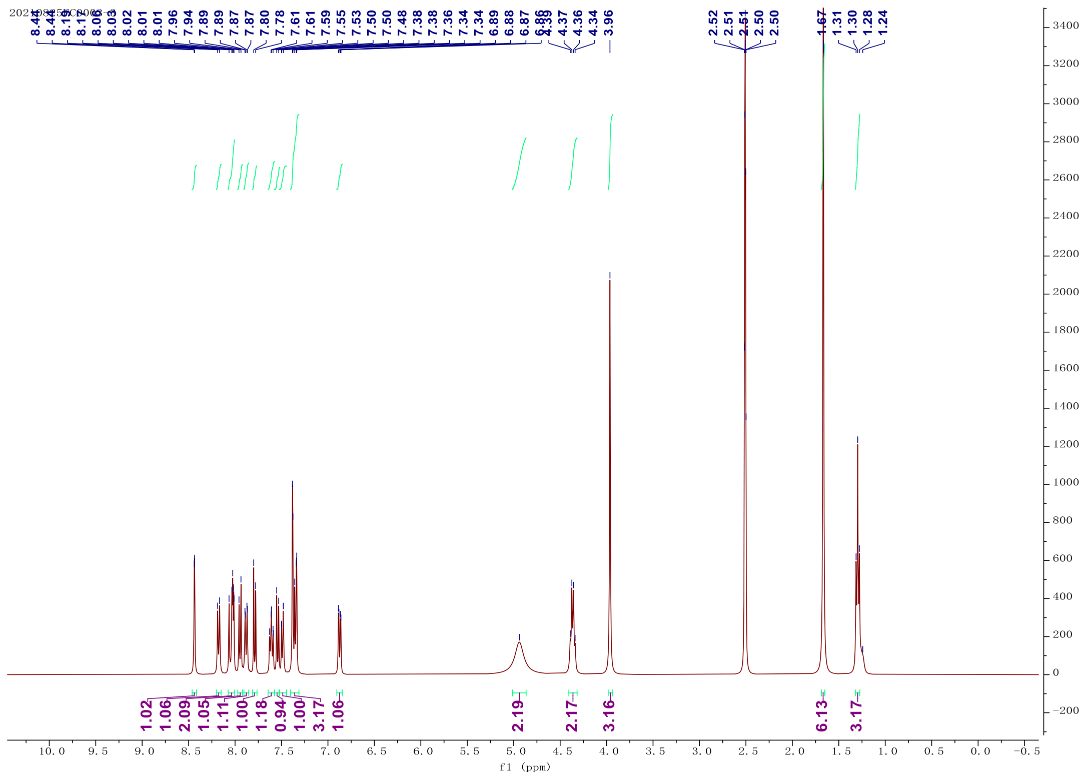

Figure S18. ^{1}H-NMR spectra of compound **A3** in DMSO-d_6.

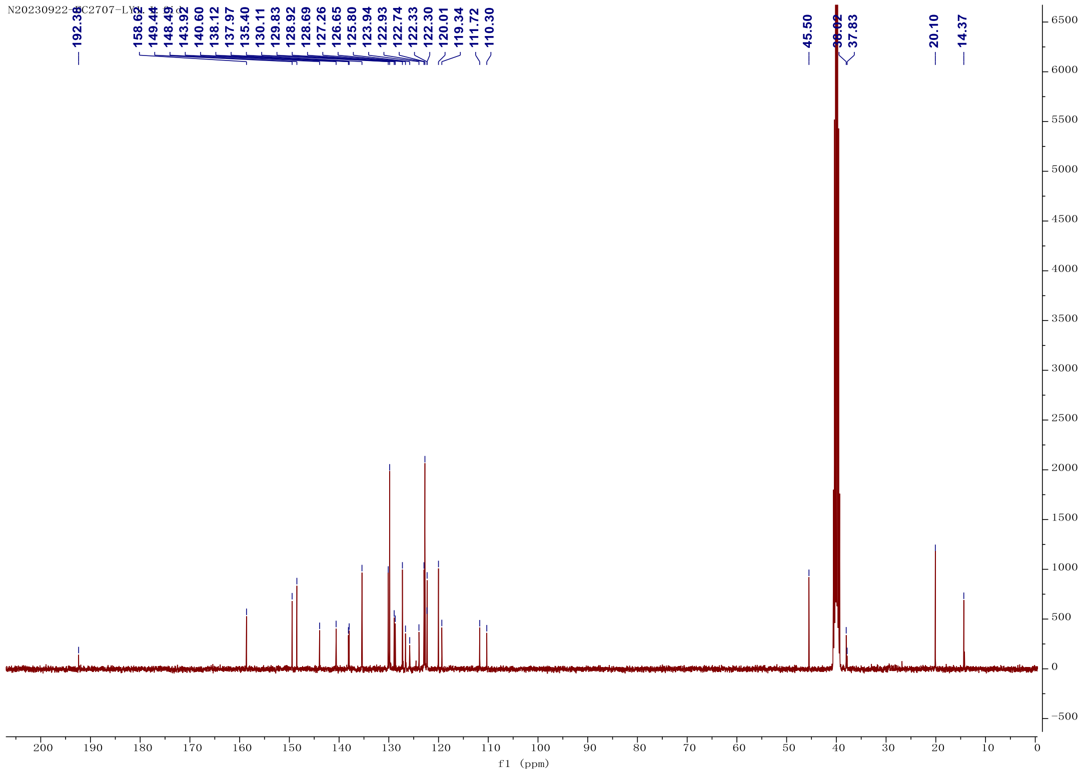

Figure S19. ^{13}C-NMR spectra of compound **A3** in DMSO-d_6.

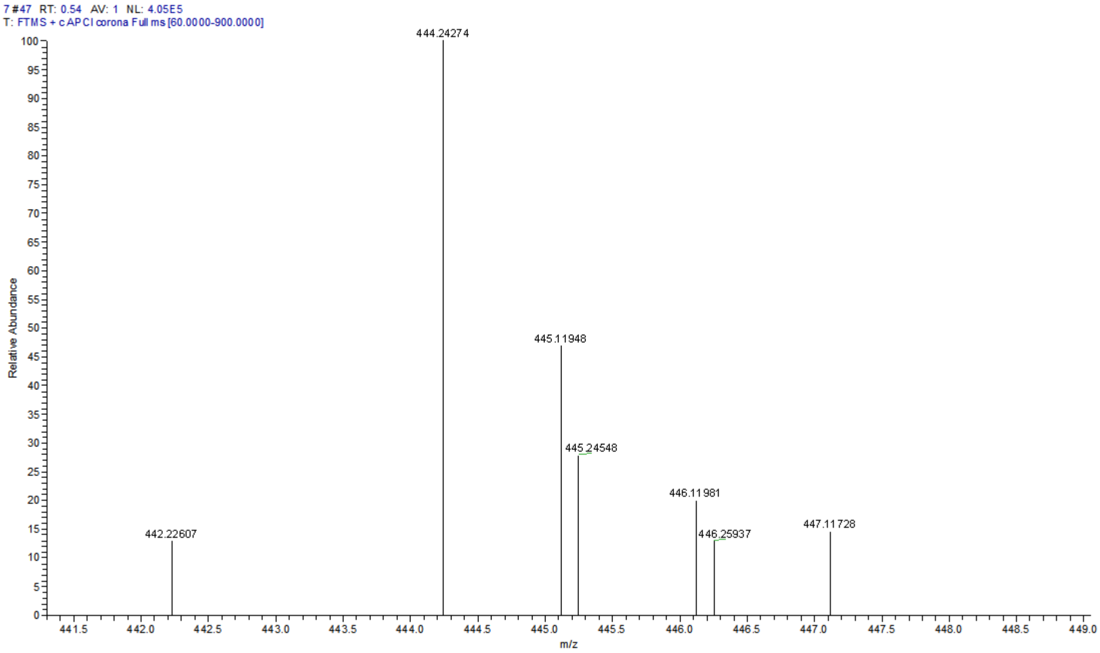

Figure S20. ESI-MS of **A3.**

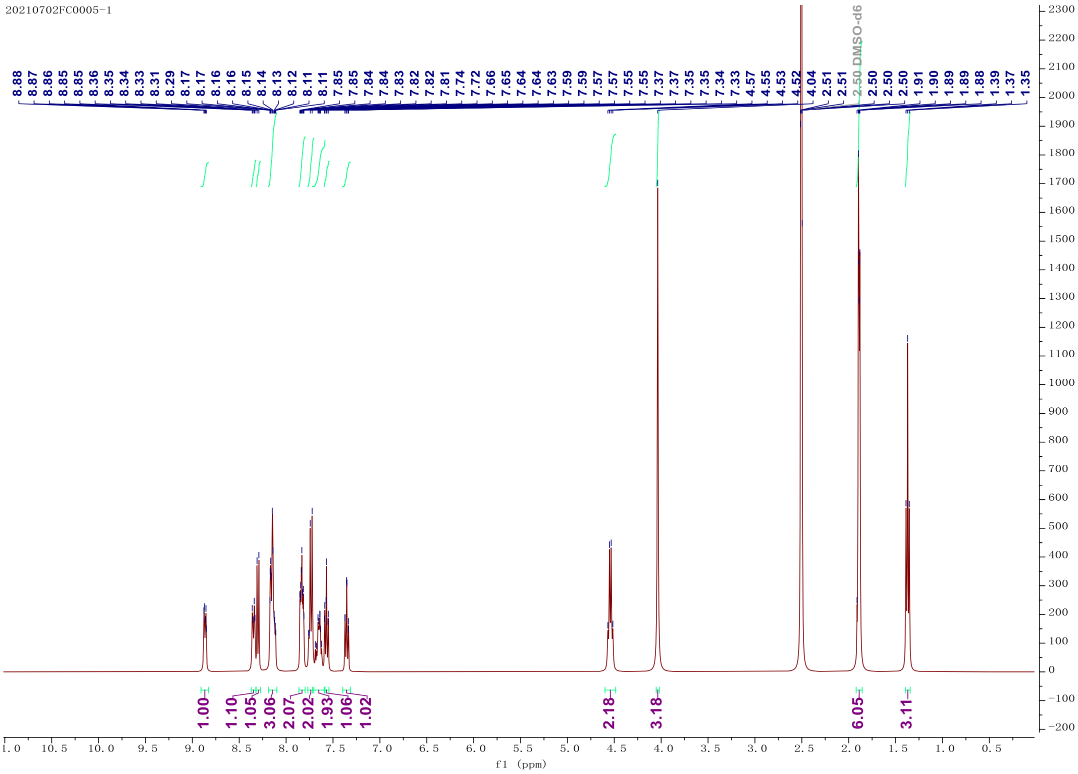

Figure S21. ^{1}H-NMR spectra of compound **A4** in DMSO-d_6.

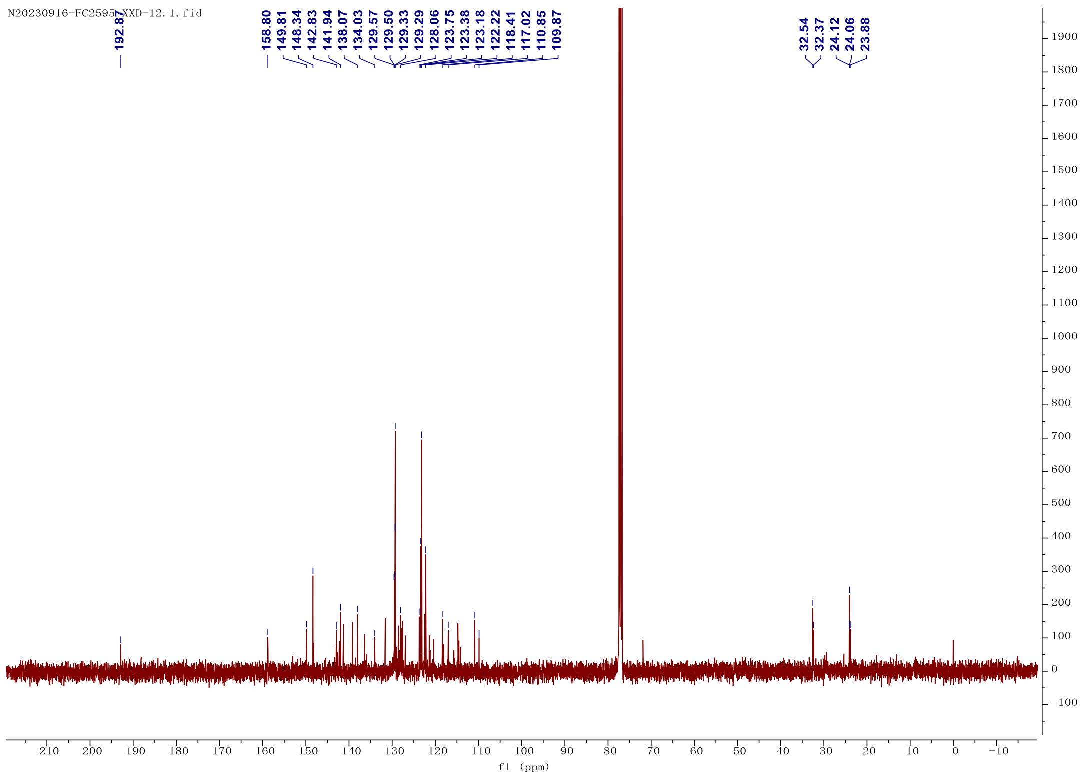

Figure S22. ^{13}C-NMR spectra of compound **A4** in $CDCl_3$.

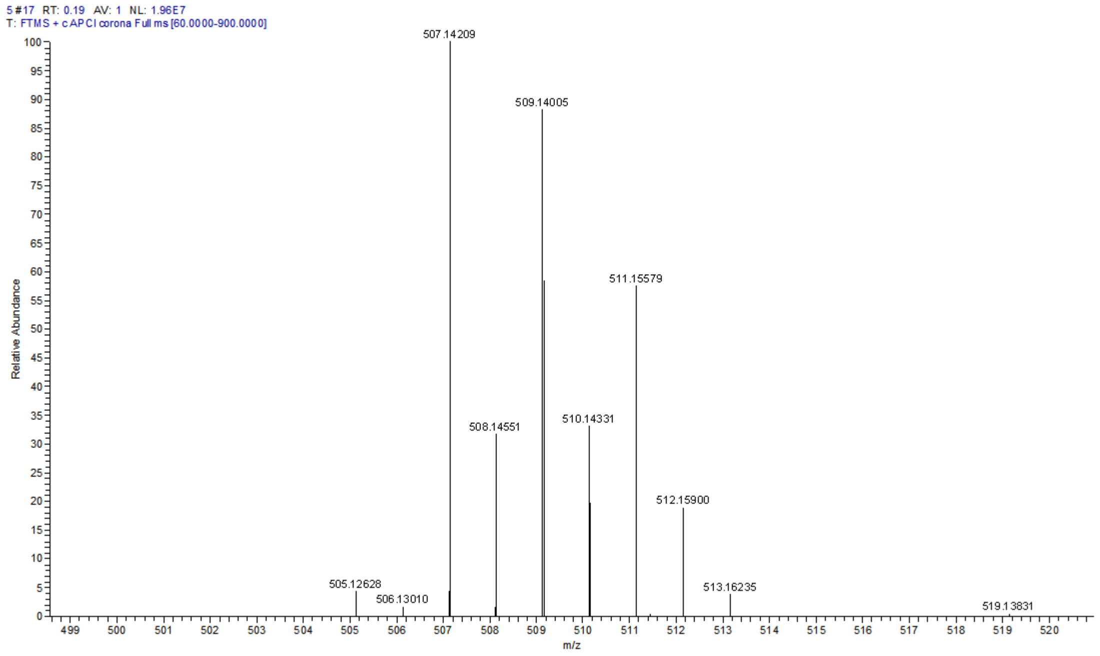

Figure S23. ESI-MS of **A4**.

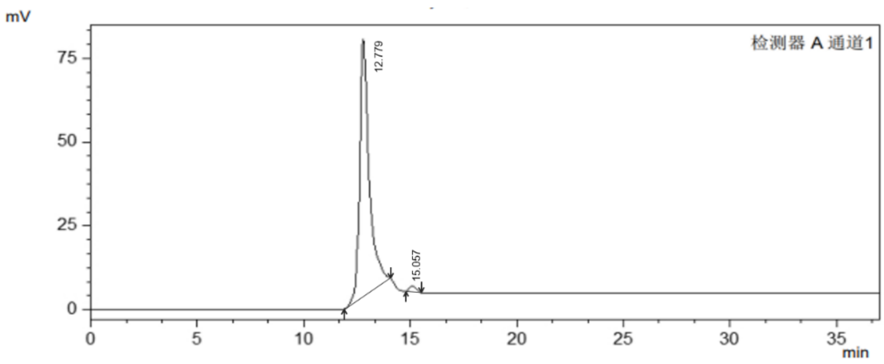

Figure S24. HPLC analysis of **A1**; Purity = 97.2%.

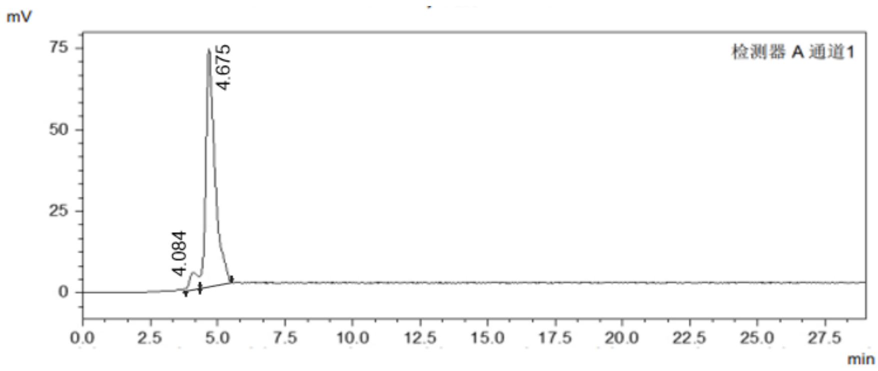

Figure S25. HPLC analysis of **A2**; Purity = 97.3%.

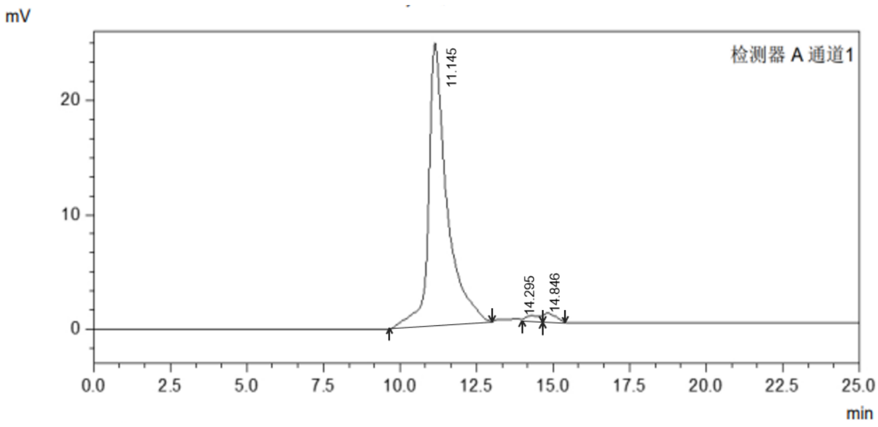

Figure S26. HPLC analysis of **A3**; Purity = 97.1%.

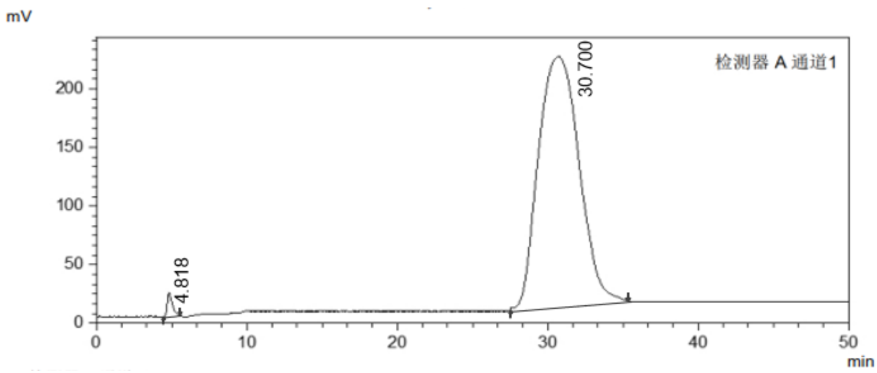

Figure S27. HPLC analysis of **A4**; Purity = 98.7%.
